# Iron-dependent apoptosis causes embryotoxicity in inflamed and obese pregnancy

**DOI:** 10.1038/s41467-021-24333-z

**Published:** 2021-06-29

**Authors:** Allison L. Fisher, Veena Sangkhae, Kamila Balušíková, Nicolaos J. Palaskas, Tomas Ganz, Elizabeta Nemeth

**Affiliations:** 1 Molecular, Cellular & Integrative Physiology Graduate Program, University of California, Los Angeles, Los Angeles, CA USA; 2 Center for Iron Disorders, David Geffen School of Medicine, University of California, Los Angeles, Los Angeles, CA USA; 3 Department of Biochemistry, Cell and Molecular Biology & Center for Research of Diabetes, Metabolism and Nutrition, Third Faculty of Medicine, Charles University, Prague, Czech Republic

**Keywords:** Apoptosis, Homeostasis, Reproductive biology, Obesity, Acute inflammation

## Abstract

Iron is essential for a healthy pregnancy, and iron supplementation is nearly universally recommended, regardless of maternal iron status. A signal of potential harm is the U-shaped association between maternal ferritin, a marker of iron stores, and risk of adverse pregnancy outcomes. However, ferritin is also induced by inflammation and may overestimate iron stores during inflammation or infection. In this study, we use mouse models to determine whether maternal iron loading, inflammation, or their interaction cause poor pregnancy outcomes. Only maternal exposure to both iron excess and inflammation, but not either condition alone, causes embryo malformations and demise. Maternal iron excess potentiates embryo injury during both LPS-induced acute inflammation and obesity-induced chronic mild inflammation. The adverse interaction depends on TNFα signaling, causes apoptosis of placental and embryo endothelium, and is prevented by anti-TNFα or antioxidant treatment. Our findings raise important questions about the safety of indiscriminate iron supplementation during pregnancy.

## Abstract (web summary)

Iron is essential during pregnancy for embryo and placental development and maternal health. However, in this study using mouse models, the authors demonstrate that excess maternal iron causes adverse embryo outcomes in pregnancies with underlying systemic inflammation.

## Introduction

Iron is an essential micronutrient required by all tissues for metabolic and cellular processes. Iron requirements increase substantially over the course of pregnancy to support growth of the fetus and placenta and for maternal erythropoietic expansion^1^. To meet these demands, both dietary iron absorption and mobilization of iron from stores increase, and this is mediated by a decrease in maternal hepcidin, the iron regulatory peptide hormone produced in the liver^2^.

Severe iron deficiency and associated anemia is detrimental for both mother and fetus^3^ which has led to the policy of universal iron supplementation even without screening for preexisting iron deficiency in most countries, including the United States. In developed countries, most women of reproductive age have adequate iron stores, and less than 25% of pregnant women have mild iron deficiency and anemia^4,5^, prompting considerations of the potential risks of indiscriminate iron supplementation.

Although iron is essential for cell function and viability, excess iron can be toxic. In iron overload conditions, high iron concentrations in plasma exceed the iron-binding capacity of transferrin, and non-transferrin-bound iron (NTBI) appears in circulation, damaging cells and tissues through the generation of reactive oxygen species^6,7^. How elevated transferrin saturation and/or the presence of NTBI in maternal circulation affects placental and fetal tissues is unknown. Pregnancies associated with iron excess include patients with hereditary hemochromatosis or β-thalassemia, the latter having well-documented complications including prematurity, abortion, and intrauterine fetal death^8,9^. However, even normal pregnancies may be exposed to excess iron and NTBI when iron-supplemented^10^, in part because of decreased maternal hepcidin allowing for highly efficient iron absorption. High iron status is associated with gestational diabetes and impaired fetal growth^11–17^. Furthermore, large epidemiological studies show a U-shaped association between maternal ferritin, a marker of iron stores, and risk of adverse outcomes such as low birthweight, stillbirth, preterm birth (<37 weeks), very preterm birth (<32 weeks’ gestation), and neonatal asphyxia^18–25^. However, these harms are not necessarily caused by iron excess as ferritin is also increased by inflammation and may not accurately reflect iron stores in the presence of inflammation or infection.

Maternal systemic inflammation is independently associated with adverse outcomes including increased risk of schizophrenia, autism, and other psychiatric disorders^26,27^. This was observed with acute inflammation during bacterial or viral infectious episodes^28,29^ and in milder chronic conditions such as obesity^30,31^. Animal models that investigated the underlying mechanisms implicated proinflammatory cytokines as a cause of abnormal fetal brain development and autism-associated behaviors in offspring^32–35^. In humans, maternal serum tumor necrosis factor α (TNFα) is elevated in pathologic pregnancies^36^ and in first trimester loss^37^. Moreover, elevated interleukin (IL) 8, TNFα, and C-reactive protein are associated with the risk of schizophrenia in offspring^38–40^.

Since high ferritin is a marker of both high iron and inflammation, it is unclear if adverse birth outcomes associated with high maternal ferritin are caused by high iron, inflammation, or a combination of both, and which pathways mediate injury to the fetus. To address these questions, we developed and examined mouse models of maternal iron excess and systemic inflammation during pregnancy and discovered an adverse synergy: only the presence of both resulted in embryotoxicity. Embryotoxicity was observed in a LPS-induced sepsis model and with diet-induced obesity. The underlying mechanism is dependent on iron-induced oxidative stress sensitizing endothelium in the placenta and embryo to TNFα-mediated apoptosis. These findings are surprising in view of the remarkable resistance of mouse models to tissue injury from massive iron overload induced by dietary or genetic manipulations. Synergistic toxicity of iron and inflammation expands our understanding of the potential effects of iron supplementation not only in pregnant women but also during other inflammatory conditions.

## Results

### Maternal iron overload and systemic inflammation synergize to cause embryotoxicity

To model iron supplementation, C57BL/6 females were fed high-iron diet (2500–5000 ppm carbonyl iron) for 1–3 weeks prior to and during pregnancy (4–6 weeks total). In addition, we used a genetic model of iron excess: C57BL/6 mice deficient in the iron regulatory hormone hepcidin (hepcidin knockout (KO)) which accumulate iron naturally on standard chow (185 ppm iron). Control iron-adequate dams were fed standard chow. Non-pregnant females were age- and diet-matched to pregnant females. In both models of iron loading, pregnant females had higher hepatic iron compared to iron-adequate dams at E15.5 and 18.5 (all *P* < 0.01) (Supplementary Fig. 1a). Serum iron was significantly elevated in hepcidin KOs but not in dietary-loaded wild-type dams which express hepcidin (Supplementary Fig. 1b). Embryos were relatively protected from iron overload on E15.5, but by E18.5, embryo liver iron stores and serum iron were significantly higher in both iron-loading models relative to iron-adequate controls (both *P* < 0.01) (Supplementary Fig. 1c, d). Of note, the severity of embryo liver iron loading was much lower than in the dams. Placenta iron concentration was increased in both iron loading groups at E15.5 and 18.5 but was more pronounced at E18.5 (both *P* < 0.001) (Supplementary Fig. 1e). Iron loading did not alter litter size (Supplementary Fig. 1f).

**Fig. 1:**
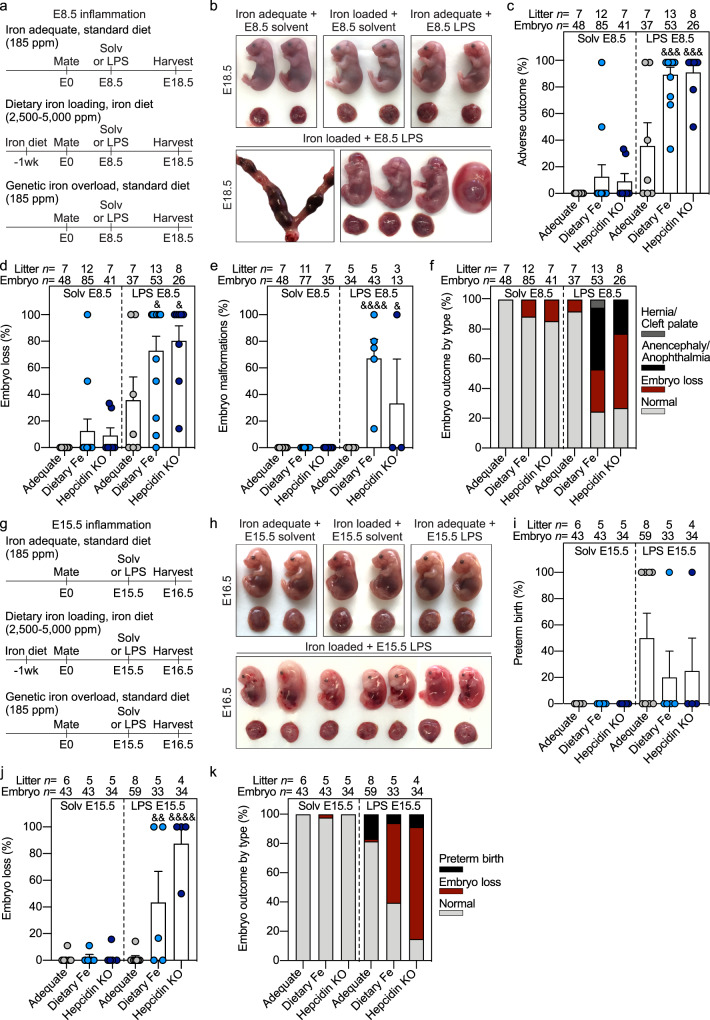
Maternal iron overload and systemic inflammation synergize to cause embryotoxicity. Dietary iron loading was achieved by feeding WT mice a high iron diet (light blue circles, 2500–5000 ppm iron) for 1–3 weeks before mating and during pregnancy. Hepcidin KO dams (dark blue circles), a model of genetic iron loading, were fed standard diet (185 ppm iron). Both dietary and genetic iron loaded pregnancies were compared to iron-adequate pregnancies (WT females fed standard diet, gray circles) under normal conditions and with maternal inflammation. **a** Maternal systemic inflammation was induced on E8.5 by a single subcutaneous injection of 0.5 μg/g LPS in the interscapular area and dams were euthanized on E18.5: **b** embryo gross morphology, **c** incidence of adverse pregnancy outcome, **d** embryo loss and **e** embryo malformation. **f** Incidence of embryo malformation by type. **g** Maternal systemic inflammation was induced on E15.5 by a single subcutaneous injection of 0.5 μg/g LPS for 24 h: **h** embryo gross morphology, **i** incidence of preterm birth and **j** embryo resorption. **k** Incidence of embryo outcome by type. **c**–**e**, **i**, **j** Error bars represent mean ± s.e.m. Statistical differences were determined by two-way ANOVA followed by Holm-Sidak method for multiple comparisons. ^&^*P* < 0.05, ^&&^*P* < 0.01, ^&&&^*P* < 0.001, ^&&&&^*P* < 0.0001. Source data are provided as a Source data file.

Acute systemic maternal inflammation was induced by a single subcutaneous injection of LPS on E8.5 or 15.5 in the three group of dams: those on iron-adequate or high-iron diet and hepcidin KOs. Control dams in each group were injected with solvent. After the E8.5 injection, embryo outcome was evaluated on E18.5 (Fig. 1a). Injection of solvent did not cause abnormalities in any groups except we observed embryo loss (fatal subcutaneous hemorrhaging) in a single litter in dietary iron-loaded dams at E18.5 (Fig. 1b–f). Thus, iron loading by itself did not cause severe embryo damage. E8.5 LPS injection in iron-adequate pregnancies caused adverse outcomes in 36% of pregnancies, involving embryo loss (embryo resorption or fatal hemorrhaging) but not embryo malformations (Fig. 1b–f). Remarkably, when dams were both iron-loaded and LPS-injected, we observed a striking synergistic effect: adverse outcomes were observed in 90% of hepcidin KO and dietary iron-loaded dams and involved embryo loss or malformations (both *P* < 0.001) (Fig. 1c), indicating that elevated maternal iron rather than the lack of hepcidin was the pathogenic factor synergizing with inflammation. Similar embryo outcomes observed in wild-type dams fed high iron diet and hepcidin KO dams fed standard diet were indicative that neither diet composition nor potential iron-related changes to maternal microbiome contributed to adverse embryo outcomes. Embryo loss was noted in 73% of dietary iron-loaded pregnancies (*P* = 0.036) and in 80% of hepcidin KO pregnancies (*P* = 0.032) (Fig. 1b, d). Embryo malformations occurred in 67% of dietary iron-loaded embryos (*P* < 0.001) and 33% of hepcidin KO embryos (*P* = 0.033) (Fig. 1b, e). Embryonic malformations most commonly included anencephaly and/or anophthalmia, and less commonly hernia or cleft palate (Fig. 1b, e, f). We observed mild decreases in E18.5 placenta weights after E8.5 LPS treatment in the iron-adequate and hepcidin KO group (Supplementary Fig. 1g). Embryo weights were mildly increased in the iron-adequate group but decreased in the dietary iron loaded group following LPS treatment (Supplementary Fig. 1h). Of note, embryo weights represent healthy and malformed embryos but exclude resorbing embryos in which weights were not accurately obtained.

Since E8.5 LPS injection caused embryo loss in iron-loaded dams, this limited the availability of embryo tissue for analysis. We thus injected LPS or solvent on E15.5 and analyzed outcomes after 24 h (Fig. 1g). Solvent injections did not cause preterm birth or embryo resorption in any groups, confirming that maternal iron loading by itself does not cause severe damage to embryos. In dams with normal iron status, E15.5 LPS injection caused preterm birth in 50% of dams, but embryos appeared normal with only 2% lethality (Fig. 1h–k). In iron-loaded dams, however, LPS injection induced much higher embryonic lethality: 43% in dietary iron-loaded dams (*P* = 0.003), and 88% in hepcidin KO dams (*P* < 0.001) (Fig. 1g–k). Similar to the E8.5 LPS treatment group, we observed mild decreases in E16.5 placental weights in the iron-adequate and hepcidin KO group after E15.5 LPS treatment (Supplementary Fig. 1i). Collectively, these data show that maternal iron excess, elicited by dietary or genetic means, potentiates the adverse effects of systemic inflammation during pregnancy.

LPS is a component of Gram-negative bacteria but numerous viral and bacterial infections during pregnancy are known to cause adverse outcomes^41,42^. We tested if high maternal iron potentiates adverse outcomes in dams injected with different pathogen-associated molecules (PAMPs). Iron-adequate wild-type and hepcidin KO dams were treated with polyinosinic:polycytidylic acid, lipoteichoic acid, pam3csk4, or flagellin on E15.5, and outcomes were analyzed on E16.5 (Supplementary Table 1). In order to study synergy with iron excess, we chose doses that were lower than those previously reported to trigger preterm birth in iron-adequate mice^43^, but most were sufficient to induce maternal inflammation comparable to the LPS group as measured by liver *Saa1* expression (Supplementary Table 1). However, these PAMPS had no obvious detectable effects on mouse pregnancy compared to the severe outcomes with LPS. We did not observe preterm birth nor adverse embryo outcomes in either iron-adequate or iron-loaded dams, but we did not evaluate subtler or delayed phenotypes such as behavioral deficits, delayed embryo loss or stillbirth at term since the pregnancies did not continue past 24 h of PAMP injection. It is also possible that different PAMPs induce different cytokine profiles. It remains to be determined whether higher PAMP doses or live infections would synergize with iron excess.

To address the underlying mechanisms of adverse synergy observed in the LPS model, we examined if maternal iron loading augments the inflammatory response in the dam, placenta, and embryo. Multiplex analysis of 32 cytokines in maternal serum showed that although 24 cytokines were induced by LPS within 6 h, the induction was similar between iron-adequate and iron-loaded dams (either dietary-loaded or hepcidin KOs), including TNFα, IL6, IL1β, and IL10 (Supplementary Fig. 2a–d). In the placenta, *Tnf* and *Il6* expression increased 6 h after LPS independently of iron status (Supplementary Fig. 2e, f). To obtain enough volume for embryo serum cytokine analysis, a separate cohort of iron-adequate and iron-loaded dams were injected with LPS on E17.5 for 6 h and embryo serum was pooled from each litter. Out of 32 cytokines measured, only IL6 and GCSF (Supplementary Fig. 2g, h) increased in embryo serum but this was independent of maternal iron status. We did not detect any increases in embryo serum TNFα, IL1β, or IL10 (Supplementary Fig. 2i–k). LPS was undetectable in embryo serum after subcutaneous injection in the dam on E17.5 (Supplementary Fig. 3a), suggesting that at least for the E15.5 time point, LPS likely did not cross the placenta to directly cause embryotoxicity.

Collectively, our data show that LPS injection induces acute, transient inflammation that is primarily restricted to the dam and placenta and is independent of maternal iron status. Thus, differential activation of maternal inflammatory pathways is not responsible for the synergistic effects observed with maternal iron excess and systemic inflammation.

We next measured maternal progesterone, as premature progesterone withdrawal triggers preterm birth in mice^44^. LPS treatment induced progesterone withdrawal within 6 h regardless of iron status (Supplementary Fig. 3b), consistent with the incidence of preterm birth observed in Fig. 1i. Additionally, since high iron is associated with oxidative stress, we measured malondialdehyde (MDA) in maternal serum. There was no difference in MDA between iron-adequate and iron-loaded groups at baseline or 6 h after LPS. MDA was elevated in iron-loaded dams 24 h after LPS treatment (Supplementary Fig. 3c), suggesting that the increase in MDA is not a causative factor leading to embryo demise but rather a consequence of embryo resorption. These data suggest that neither maternal progesterone changes nor maternal systemic oxidative stress are responsible for the adverse synergy between iron excess and inflammation.

### Adverse synergy between maternal iron excess and inflammation targets placental and embryo endothelium to cause its apoptosis

We performed a screen of activated cell death pathways in placentas and embryo tissues. No iron-dependent difference was observed in protein levels of pyroptotic markers IL1β or cleaved caspase-1, mRNA for the NLRP3 component of inflammasome, lipid peroxidation marker MDA, or putative ferroptosis marker *Ptgs2* (Supplementary Fig. 3d–k). To assess oxidative stress, we measured expression of the NRF2 target gene *Nqo1*, as a marker of antioxidant pathway activation. *Nqo1* expression in embryo liver did not differ between iron-adequate and loaded groups (Supplementary Fig. 3l). In whole placentas, *Nqo1* was moderately increased in the iron-loaded group 6 and 24 h after LPS treatment (both *P* < 0.001) (Supplementary Fig. 3m). Similarly, *Hmox1* was moderately increased at baseline (*P* = 0.026) and 24 h after LPS treatment (*P* < 0.001) (Supplementary Fig. 3n), suggesting that iron loading and inflammation synergize to induce placental oxidative stress.

**Fig. 2:**
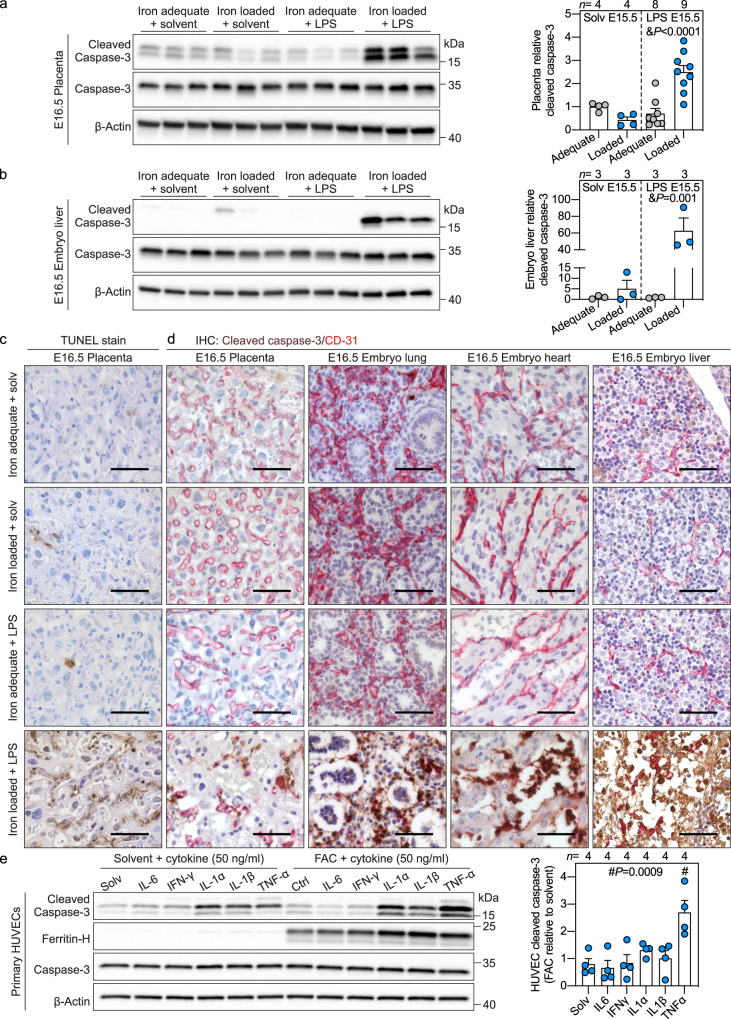
Adverse synergy between maternal iron excess and inflammation targets placental and embryo endothelium to cause its apoptosis. **a**–**d** Maternal systemic inflammation was induced in iron-adequate (gray circles) and iron-loaded (blue circles) dams on E15.5 by a single subcutaneous injection of 0.5 μg/g LPS for 24 h. Western blot (left) and quantitation (right) of **a** whole placenta and **b** embryo liver for apoptotic marker cleaved caspase-3 normalized to β-actin (*n* = 3-9/group). **c** TUNEL stain of placental sections. Representative images of *n* = 3 sections/group. **d** Immunohistochemistry for cleaved caspase-3 (brown) and endothelial marker CD31 (red) in paraffin-embedded placenta (*n* = 3), embryo lung (*n* = 5), heart (*n* = 5), and liver (*n* = 5) sections. Scale bar= 50 μm. **a**–**d** Embryo and placentas were randomly selected for analysis. **e** Primary HUVECs were treated with 100 μM ferric ammonium citrate (FAC) for 24 h prior to being stimulated with IL6, IFNγ, IL1α, IL1β, or TNFα (all 50 ng/ml) in solvent or FAC-supplemented media for 16 h. Western blot (left) and quantitation (right) for cleaved caspase-3 normalized to β-actin, representative image of *n* = 4 independent experiments. Ferritin-H is a marker of cellular iron loading. **a**, **b**, **e** Error bars represent mean ± s.e.m. Statistical differences were determined by two-way ANOVA (denoted by &) or one-way ANOVA (denoted by #) followed by Holm-Sidak method for multiple comparisons. *P*-values are indicated in each figure panel. Source data are provided as a Source data file.

To examine apoptosis, we measured cleaved caspase-3, a canonical marker of programmed cell death. Placental cleaved caspase-3 was not increased after 6 h (Supplementary Fig. 3o), however, 24 h after maternal LPS injection, cleaved caspase-3 increased in placenta and embryo liver in iron-loaded, inflamed pregnancies (Fig. 2a, b). TUNEL staining of placental sections confirmed increased cell death in the labyrinth layer only when the dam was both iron-loaded and LPS-injected (Fig. 2c), with a distribution pattern suggesting endothelial cell death. Co-staining of placental and whole embryo sections for cleaved caspase-3 and endothelial marker CD31 demonstrated colocalization of the two proteins in the placenta, embryo lung, heart, liver, and umbilical cord only in iron-loaded, inflamed pregnancy (Fig. 2d, Supplementary Fig. 4a), indicating that the adverse synergy targets the placental and embryo endothelial cells and causes their apoptosis. In some embryos, however, we observed widespread apoptosis in organs, and thus cannot exclude the possibility that cell types other than endothelial cells are also susceptible to the adverse interaction between iron and inflammation.

To identify the mechanisms leading to endothelial apoptosis, we used human umbilical vein endothelial cells (HUVEC), primary fetal endothelial cells from the umbilical cord, a tissue that was affected by endothelial apoptosis in the mouse model. HUVECs were treated with ferric ammonium citrate (FAC) to induce iron loading (confirmed by ferritin immunoblotting), then stimulated with cytokines IL6, IFNγ, IL1α, IL1β, and TNFα (Fig. 2e). All of these except IL1α were elevated in maternal circulation 6 h after LPS treatment, independently of the dam iron status. In HUVECs, IL6 and IFNγ did not induce cleaved caspase-3 in either solvent or iron-treated cells, whereas IL1α and IL1β did induce cleaved caspase-3 but similarly in solvent or iron-treated groups. Only the induction of cleaved caspase-3 by TNFα was strongly potentiated by iron loading (*P* < 0.001, Fig. 2e). Direct LPS treatment did not induce HUVEC cleaved caspase-3 (Supplementary Fig. 4b). The specificity of the interaction between iron and TNFα was demonstrated by treating HUVECs with other metals. Only iron but not copper or zinc potentiated TNFα induction of cleaved caspase-3 (Supplementary Fig. 4c). Furthermore, co-treating HUVECs with FAC and iron chelator deferoxamine prevented TNFα-mediated apoptosis (Supplementary Fig. 4d). Time course of apoptosis in solvent and FAC-treated HUVECs indicated that cleaved caspase-3 expression was potentiated in iron-laden cells as early as 6 h after TNFα treatment (*P* < 0.0001) (Supplementary Fig. 4e).

Collectively, our data show that iron accumulation sensitizes endothelial cells to TNFα-induced cell death.

### Synergistic toxicity of iron and inflammation is mediated by maternal TNFα

Pathways leading to apoptosis are categorized into the extrinsic or death receptor pathway, and the intrinsic or mitochondrial pathway^45^, but both lead to caspase-3 cleavage. We did not detect any iron-dependent differences in cleaved caspase-9 or mitochondrial oxidative phosphorylation complexes in HUVECs treated with FAC and TNFα, or in placenta lysates from the iron-loaded, inflamed group (Supplementary Fig. 5a, b), further suggesting that iron excess alters the extrinsic apoptotic pathway.

**Fig. 3:**
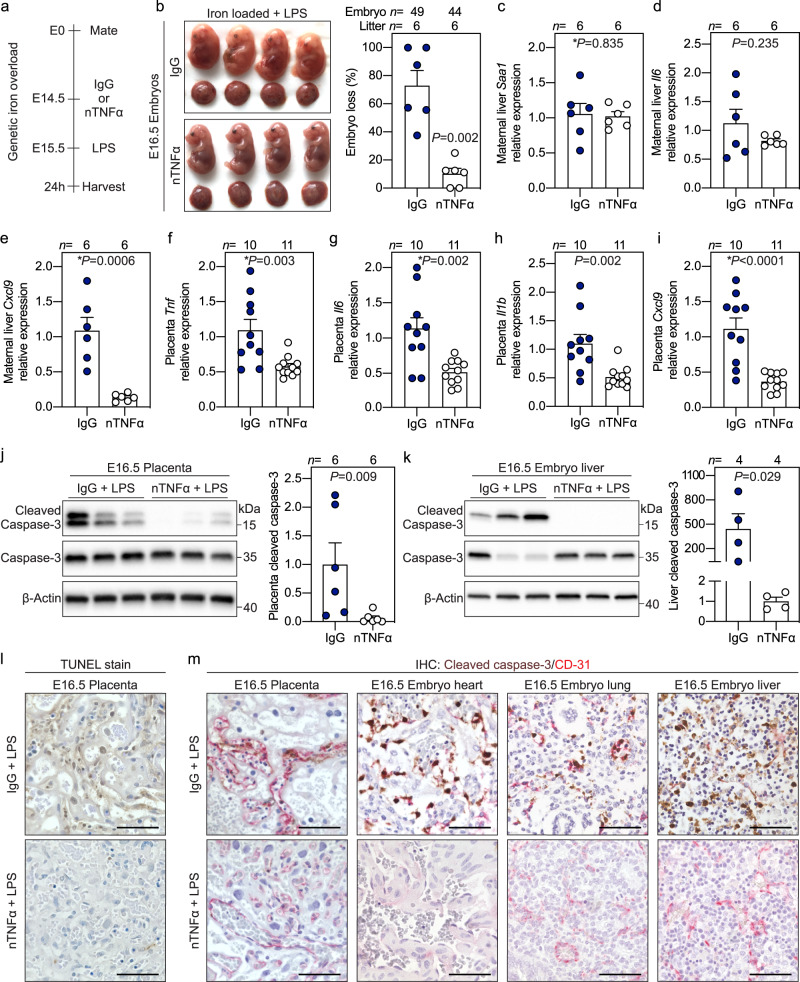
Synergistic toxicity of iron and inflammation is mediated by maternal TNFα. **a** Iron-loaded hepcidin KO dams were treated intravenously via the retroorbital sinus with 500 μg neutralizing TNFα antibody (nTNFα, white circles) or isotype control IgG (IgG, blue circles) neutralizing trinitrophenol 15 h prior to subcutaneous injection of LPS (0.5 μg/g) on E15.5 for 24 h. **b** Gross morphology and incidence of resorbing embryos. **c**–**e** Maternal liver expression of inflammatory markers *Saa1*, *Il6*, and *Cxcl9* normalized to *Hprt*. **f**–**i** Placental mRNA expression of inflammatory markers *Tnf*, *Il6*, *Il1b*, and *Cxcl9* normalized to *Rpl4*. **j**, **k** Western blots and quantitation of whole placenta or embryo liver lysates for cleaved caspase-3 expression normalized to β-actin. **l** TUNEL stain of placental sections. **m** Immunohistochemistry for cleaved caspase-3 (brown) and CD31 (red) in paraffin-embedded placenta, embryo heart, lung, and liver. **l**, **m** Representative images of *n* = 3 sections/group. Scale bar = 50 μm. **f**–**m** Embryo and placentas were randomly selected for analysis. **c**–**k** Error bars represent mean ± s.e.m. Statistical differences between groups were determined by two-tailed Mann–Whitney *U* or two-tailed Student’s *t*-test (denoted by *). *P*-values are indicated in each figure panel. Source data are provided as a Source data file.

We examined the contribution of TNFα in mediating embryo demise in vivo by treating pregnant iron-loaded hepcidin KO dams with TNFα-neutralizing antibody or control IgG antibody targeting trinitrophenol, prior to E15.5 LPS treatment (Fig. 3a). Importantly, in contrast to the control IgG group where LPS caused a high rate of embryo resorption, neutralizing TNFα nearly completely prevented embryo resorption and demise (*P* = 0.002, Fig. 3b). The specificity of TNFα-neutralizing antibody was confirmed in HUVECs: in iron-loaded cells, treatment with TNFα-neutralizing antibody prevented cleaved caspase-3 induction by TNFα but not IL1α or IL1β (Supplementary Fig. 6a). In pregnant hepcidin KO mice, neutralizing TNFα in maternal circulation had no effect on LPS-induced hepatic expression of serum amyloid A1 (*Saa-1*), *Il6*, *Cxcl2*, *Ccl2*, or *Il1b*, but reduced *Cxcl9* expression (Fig. 3c–e, Supplementary Fig. 6b–d). This suggests that except for *Cxcl9*, the tested biomarkers are largely independent of TNFα. Thus, anti-TNFα therapy improved embryo outcomes despite the presence of systemic maternal inflammation from TNFα-independent cytokines. Placentas from dams treated with anti-TNFα therapy had lower expression of *Tnf*, *Il6*, *Il1b*, and *Cxcl9* (all *P* < 0.01) (Fig. 3f–i), showing that anti-TNFα therapy attenuates placental inflammation. Importantly, neutralizing TNFα prevented placenta and embryo apoptosis as reflected by reduced caspase-3 cleavage in placentas (*P* = 0.009) and embryo livers (*P* = 0.029), and decreased TUNEL staining in placental sections (Fig. 3j–l). Co-staining shows that anti-TNFα prevented apoptosis of endothelial cells in the placenta, embryo heart, lung, and liver (Fig. 3m). These data confirm that maternal TNFα is the pathogenic factor causing endothelial apoptosis and embryo demise. Of note, we have not formally tested the role of TNFα-mediated apoptosis after E8.5 LPS injection, and it remains to be determined whether the same underlying mechanism leads to embryo demise even at this early time-point, when placenta is not fully formed and vascularized.

### Maternal iron loading promotes oxidative stress in placental endothelial cells

**Fig. 4:**
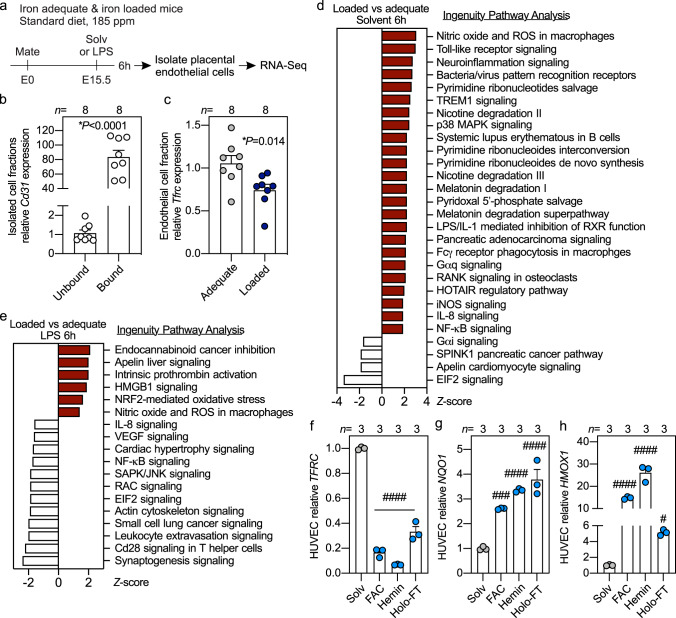
Maternal iron loading promotes oxidative stress in placental endothelial cells. **a** Iron-adequate and iron-loaded dams were treated with solvent or LPS (0.5 µg/g) on E15.5 for 6 h. Placentas from solvent and LPS-treated mice were collected for endothelial cell isolation by magnetic separation using Cd31 beads and total RNA was analyzed by RNA-Seq. **b** *Cd31* expression relative to *Rpl4* in the Cd31-unbound (non-endothelial cells) and Cd31-bound (endothelial cells) fractions. **c** Endothelial cell expression of iron importer *Tfrc* relative to *Rpl4* from iron-adequate (light gray circles) and iron-loaded (dark blue circles) placentas. **b**, **c** Placenta cells isolated from *n* = 8 dams. **d**, **e** RNA-Seq Ingenuity Pathway Analysis of significantly downregulated (white bars) and enriched (red bars) genes (*Z*-score < and > than ±1.5) from placental endothelial cells comparing hepcidin KO and WT mice after **d** solvent and **e** LPS injection. **f**–**h** Primary HUVECs were treated with solvent (gray circles), or different forms of iron (light blue circles) including ferric ammonium citrate (100 µM, FAC), hemin (20 µM), or holo-ferritin (Ft, 2 mg/ml) for 40 h and analyzed for **f** *TFRC* **g** *NQO1* and **h** *HMOX1* expression. *n* = 3 independent experiments. **b**, **c**, **f**–**h** Error bars represent mean ± s.e.m. Statistical differences were determined by two-tailed Mann–Whitney *U*, two-tailed Student’s *t*-test (denoted by *, *P*-values are indicated in each figure panel), or one-way ANOVA followed by Holm-Sidak method for multiple comparisons (denoted by ^#^*P* < 0.05, ^###^*P* < 0.001, ^####^*P* < 0.0001). Source data are provided as a Source data file.

To determine why endothelial cells from iron-loaded pregnancies are more susceptible to inflammation-induced apoptosis, we performed RNA-Seq of isolated placental endothelial cells from iron-adequate wild-type and iron-loaded hepcidin KO dams 6 h after solvent or LPS injection (Fig. 4a). Isolation of endothelial cells was confirmed by 80-fold enrichment of *Cd31* mRNA (*P* < 0.0001) (Fig. 4b). Placental endothelial cells from hepcidin KO compared to wild-type dams had lower expression of iron-importer transferrin receptor (*Tfrc*, *P* = 0.014), indicative of cellular iron loading (Fig. 4c). Ingenuity pathway analysis of differentially expressed genes showed upregulation of reactive oxygen species and canonical inflammatory signaling pathways in endothelial cells from hepcidin KO dams compared to wild-type dams (Fig. 4d). After LPS treatment, endothelial cells from hepcidin KO dams compared to LPS-treated WT dams showed further enrichment in genes in oxidative stress pathways, namely reactive oxygen species, Nrf2-oxidative stress, and Hmgb1 signaling (Fig. 4e). This agrees with the finding that whole placenta oxidative stress was increased at baseline and after LPS treatment (Supplementary Fig. 3m, n). We treated HUVECs with NTBI, heme, and iron-rich ferritin to induce iron accumulation, which was confirmed by the reduction in *TFRC* (*P* < 0.0001) (Fig. 4f). All forms of iron caused over 2-fold induction in *NQO1* and over 4-fold induction in *HMOX1* expression compared to solvent-treated cells (Fig. 4g, h), indicative of oxidative stress. These data suggest that iron loading sensitizes endothelial cells to injury through oxidative stress and enhanced inflammatory signaling.

### Synergistic toxicity of iron and inflammation is prevented by α-Tocopherol treatment

**Fig. 5:**
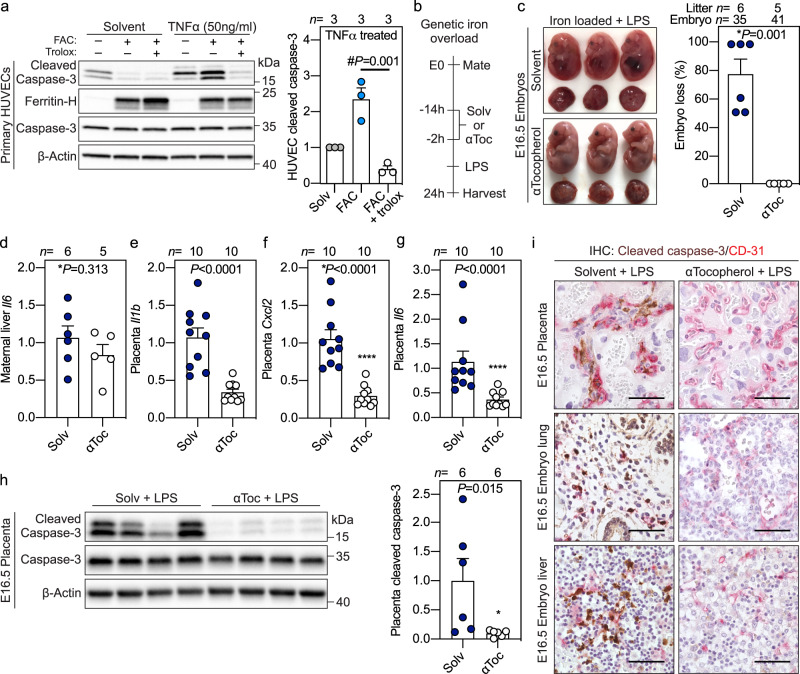
Synergistic toxicity of iron and inflammation is prevented by αTocopherol treatment. **a** Primary HUVECs were treated with solvent (gray gircles), 100 μM ferric ammonium citrate (FAC) (light blue circles), or 100 μM FAC with vitamin E analog Trolox (300 μM, white circles) for 24 h prior to being stimulated with TNFα (50 ng/ml) for 16 h. Western blot (left) and quantitation (right) of cleaved caspase-3 normalized to β-actin (representative image of *n* = 3 independent experiments). **b**–**i** Hepcidin KO dams were treated with subcutaneous solvent (dark blue circles) or αTocopherol (αToc: vitamin E, white circles) 14 and 2 h prior to LPS treatment on E15.5 for 24 h: **c** embryo gross morphology and incidence of resorbing embryos; **d** maternal liver *Il6* expression relative to *Hprt* from *n* = 6 dams; **e**–**g** placental *Il1b*, *Cxcl2*, and *Il6* expression relative to *Rpl4* from *n* = 10 placentas. **h** Western blot (left) and quantitation (right) of cleaved caspase-3 in whole placentas normalized to β-actin. *n* = 6 placentas/group. **i** Immunohistochemistry for cleaved caspase-3 (brown) and CD31 (red) in paraffin-embedded placenta, embryo lung and liver (representative image of *n* = 3 sections/group). Scale bar = 50 μm. **a**, **c**–**h** Error bars represent mean ± s.e.m. **e**–**i** Embryo and placentas were randomly selected for analysis. Statistical differences were determined by two-tailed Mann–Whitney *U*, two-tailed Student’s *t*-test (denoted by *) or one-way ANOVA followed by Holm-Sidak method for multiple comparisons (denoted by #). *P*-values are indicated in each figure panel. Source data are provided as a Source data file.

We tested the contribution of oxidative stress in iron-dependent cell death in HUVECs by treating iron-laden cells with vitamin E analog Trolox prior to TNFα stimulation. Iron loading potentiated TNFα-mediated apoptosis, which was rescued by Trolox pretreatment (Fig. 5a). To confirm the role of oxidative stress in mediating embryo demise in vivo, we treated pregnant iron-loaded hepcidin KO dams with antioxidant α-tocopherol, the most abundant isomer of vitamin E. Hepcidin KO dams were treated with subcutaneous α-tocopherol (100 µg/g) 14 and 2 h prior to LPS injection on E15.5 (Fig. 5b). Maternal pretreatment with α-tocopherol was completely protective against embryo demise (*P* = 0.001) (Fig. 5c). α-Tocopherol had no effect on maternal inflammation as shown by comparable hepatic expression of *Il6*, *Il1b*, *Cxcl2*, *Cxcl10*, *Ccl2*, and *Cxcl9* (Fig. 5d, Supplementary Fig. 6e–i). Instead, maternal α-tocopherol treatment attenuated LPS-induced inflammation in the placenta, reflected by lower expression of *Il1b*, *Cxcl2*, *Il6*, *Cxcl1*, *Ccl2*, and *Cxcl9* (Fig. 5e–g, Supplementary Fig. 6j-l). Notably, maternal α-Tocopherol treatment reduced expression of placental cleaved caspase-3 (*P* = 0.015), and prevented endothelial apoptosis in the placenta, embryo lung, and liver (Fig. 5h, i). Collectively, these data show that oxidative stress mediates endothelial apoptosis and embryotoxicity in iron-loaded and inflamed pregnancies.

### Iron supplementation potentiates embryonic malformation and placental apoptosis in a mouse model of Western diet-induced obesity

**Fig. 6:**
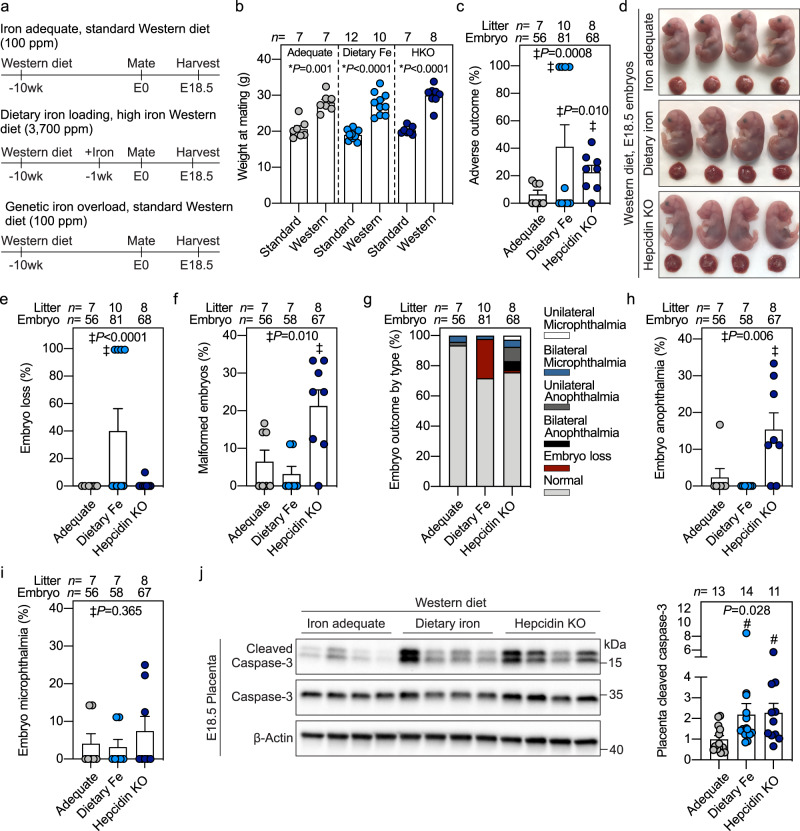
Iron supplementation potentiates embryonic malformation and placental apoptosis in a mouse model of Western diet-induced obesity. **a** Starting at 3 weeks of age, wild-type C57BL/6 (iron-adequate) or hepcidin KO (genetic iron overload, dark blue circles) females were fed an iron-adequate Western diet (100 ppm iron) for 8 weeks. After 8 weeks, half of the WT mice were switched to a high-iron Western diet (3700 ppm carbonyl iron, dietary iron loading, light blue circles) for 1–3 weeks, while the other mice continued on the iron-adequate Western diet (gray circles). Mice were mated and continued their respective diets during pregnancy. Iron adequate dams were compared to dietary or genetic iron-loaded dams. **b** Weight of female mice at mating fed a Western diet compared to those fed non-obesogenic diet. **c** Incidence of E18.5 embryos with adverse outcomes in obese pregnancies. **d** Embryo gross morphology and **e** incidence of embryos with subcutaneous hemorrhaging or **f** malformations. **g** Incidence of embryo malformations categorized by type. Incidence of embryo **h** anophthalmia and **i** microphthalmia. **j** Western blot (left) and quantitation (right) of cleaved caspase-3 in whole placentas normalized to β-actin. Representative blot of *n* = 11–14 randomly selected placentas/group. **b**, **c**, **e**, **f**, **h**–**j** Error bars represent mean ± s.e.m. Statistical differences were determined by two-tailed Student’s *t*-test (denoted by *), one-way ANOVA on ranks followed by Dunn’s method for multiple comparisons (denoted by #), or Fisher’s exact test (indicated by ‡). *P*-values are indicated in each figure panel. Source data are provided as a Source data file.

To investigate if maternal iron excess adversely interacts with chronic inflammation during pregnancy, we used a mouse model of diet-induced obesity in WT and hepcidin KOs (Fig. 6a). WT C57BL/6 females were fed an iron-adequate Western diet (100 ppm iron) for 8 weeks, then half of the mice continued on the same Western diet (iron-adequate group), and the other half were switched to a high-iron Western diet (3700 ppm carbonyl iron) for 1–3 weeks (dietary iron loading group), then mated and fed the same diet in pregnancy. The third group (genetic iron loading) consisted of hepcidin KO females maintained on the iron-adequate Western diet for the same duration. Dams in all three Western diet groups weighed ~40% more at mating than their respective controls fed non-obesogenic standard diet or high iron diet (Fig. 6b). At E18.5, compared to iron-adequate dams, hepcidin KOs were most iron-loaded with >20-fold higher liver iron and >2-fold higher serum iron (both *P* < 0.001), whereas dietary iron-loaded dams had ~6-fold higher liver iron (*P* = 0.038) but no increase in serum iron (Supplementary Fig. 7a, b). Placental iron increased in both dietary-loaded and hepcidin KO groups (*P* < 0.05) (Supplementary Fig. 7c). Embryos were completely protected from iron loading and had liver iron and serum iron concentrations comparable to embryos from iron-adequate dams (Supplementary Fig. 7d, e).

Examination of embryos for gross malformations demonstrated higher incidence of adverse embryo outcomes when both maternal iron excess and obesity were present. Obesity in iron-adequate dams caused embryo complications including eye malformations in only ~6% of embryos (Fig. 6c–i). Dietary iron loading of obese dams caused complications in 41% of pregnancies (Fig. 6c), including embryo loss characterized by resorption or fatal subcutaneous hemorrhaging (*P* < 0.001) (Fig. 6d, e), with eye malformations affecting <5% of embryos (Fig. 6f–i). Obesity in the most iron-loaded hepcidin KO dams caused complications in 22% of pregnancies (*P* = 0.010) (Fig. 6c), and prominently caused eye malformations (*P* = 0.010, Fig. 6d–i). Interestingly, only anophthalmia was worsened by genetic iron loading of obese dams (*P* = 0.006), whereas microphthalmia was similar between the groups (Fig. 6g–i). Like our acute LPS model, we observed an increase in placental cleaved caspase-3 in iron-loaded obese dams (*P* = 0.028) (Fig. 6j), suggesting similarities in the pathogenic mechanism between our acute and chronic inflammation models. It remains to be determined whether additional, less visible embryo abnormalities, are also induced by iron excess in obese pregnancy.

### Neutralizing maternal TNFα is protective against embryonic malformation induced by the combination of high maternal iron and Western diet

**Fig. 7:**
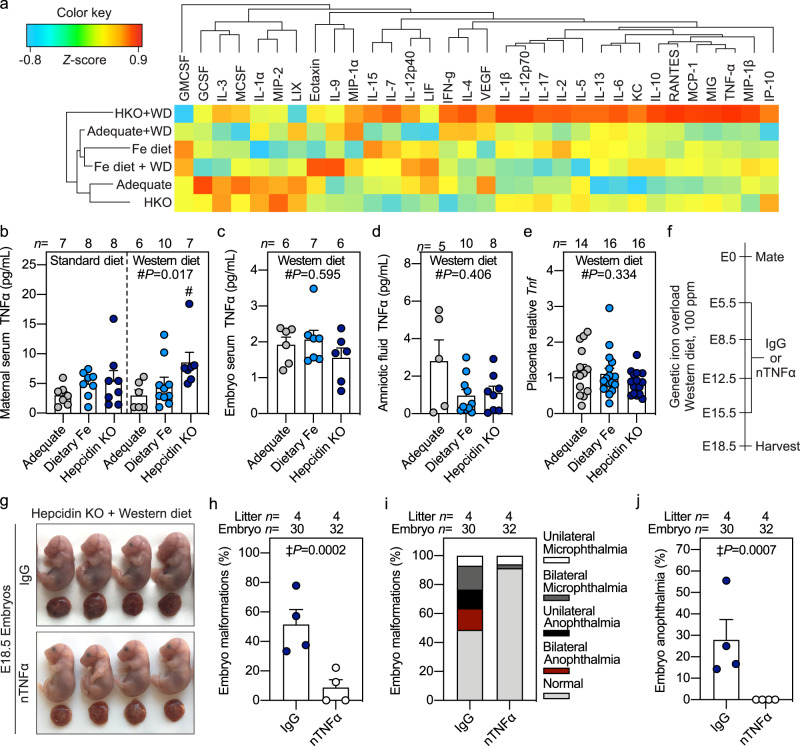
Neutralizing maternal TNFα is protective against embryonic malformation induced by the combination of high maternal iron and Western diet. **a** Clustering analysis of 32-plex cytokine panel in maternal serum from iron-adequate (gray circles), dietary iron-loaded (Fe diet, light blue circles), and genetically iron-loaded hepcidin KO (HKO, dark blue circles) dams fed standard or Western diet (WD). Color key indicates *Z*-score for each cytokine. **b**–**e** E18.5 TNFα measurements in **b** maternal serum, **c** embryo serum pooled from each litter, **d** pooled amniotic fluid, and **e** placental *Tnf* gene expression. Placentas were randomly selected for analysis. **f** Hepcidin KO females were fed Western diet (100 ppm iron) starting at 3 weeks of age and were mated after 9 weeks. Pregnant dams received intravenous injections of neutralizing TNFα antibody (white circles) or isotype IgG (dark blue circles) targeting trinitrophenol as a control (250 µg/injection) on E5.5, E8.5, E12.5, and E15.5, and embryo outcome was evaluated at E18.5. **g** Embryo gross morphology. **h**–**j** Incidence of eye malformations. **b**–**e**, **h**, **j** Error bars represent mean ± s.e.m. Statistical differences were determined by one-way ANOVA on ranks followed by Dunn’s method for multiple comparisons (denoted by #) or two-tailed Fisher’s exact test (indicated by ‡). *P*-values are indicated in each figure panel. Source data are provided as a Source data file.

We evaluated if maternal exposure to high iron while on an obesogenic diet affected inflammation, as both obese humans and animal models are reported to have elevated TNFα and IL6^46–48^. Clustering analysis of Luminex 32-plex cytokine panel in maternal serum from obese and lean mouse pregnancies with and without iron loading identified the most severe group—obese hepcidin KO dams—as having a distinct cytokine profile compared to the other groups and showed increases in TNFα and related cytokines (Fig. 7a). Elevated TNFα levels were confirmed in obese hepcidin KO dams (*P* = 0.017), but not in others (Fig. 7b). No differences in TNFα levels were found in sera from their embryos or amniotic fluid, or in placental *Tnf* expression (Fig. 7c–e), suggesting that maternal TNFα is the driver of embryonic complications. Thus, we treated hepcidin KO dams maintained on Western diet with neutralizing TNFα antibody or isotype control IgG antibody throughput pregnancy (E5.5, E8.5, E12.5, and E15.5) and analyzed embryo outcome at E18.5 (Fig. 7f). We observed eye malformations in embryos from obese hepcidin KO dams treated with IgG control, whereas neutralizing TNFα reduced the incidence of embryo eye malformations including a reduction in anophthalmia (*P* < 0.001) (Fig. 7g–j), confirming the role of maternal TNFα in mediating adverse embryo outcomes in iron-loaded, obese pregnancies.

## Discussion

While the WHO recommends that all pregnant women take daily iron supplements^49^, the US Preventive Services Task Force concluded that there are insufficient data to assess the balance of benefits and harms of iron supplementation during pregnancy^50^. In developed countries, most women have sufficient iron stores, but women are still advised to take 30–60 mg elemental iron daily starting at the first prenatal visit^3^, even without screening for iron deficiency. We recently showed that the maternal homeostatic adaptations during pregnancy include suppression of the iron regulatory hormone hepcidin^2^, which would promote the rapid absorption of iron supplements, potentially exposing pregnant women to iron excess.

Maternal serum ferritin, a marker of body iron stores, shows a U-shaped risk curve where both low and high ferritin are associated with adverse pregnancy outcomes. However, ferritin is also induced by inflammation and does not accurately reflect iron stores in the setting of inflammation or infection. Thus, the association of high ferritin with adverse outcomes could reflect the pathogenic role of iron excess or inflammation, or the combination of both. We addressed this question and explored the relevant mechanisms using mouse models and identified an interaction between iron and inflammation. Our study demonstrates iron-dependent apoptotic injury in inflamed pregnancy and the potential consequences to embryo health and development when dams are concurrently exposed to iron overload and inflammation.

The interaction between iron overload and inflammation is surprising because uninflamed mice are resistant to tissue injury from even massive iron overload. We analyzed the consequences of pure iron excess in pregnancy by modeling two modes of iron loading. One model involved feeding mice high iron diet during pregnancy to mimic the common practice of iron supplementation of human pregnancy. The range of iron content in the mouse diet was proportionally similar to what is recommended to pregnant humans. In pregnant women, depending on the country and woman’s iron status, iron supplementation ranges from 30 to 200 mg of elemental iron per day^51,52^, up to 20-fold more than the daily dietary iron intake of ~10 mg^53^. In our purified diets, the iron content varied from 2500–5000 ppm iron, which is ~13–27-fold more than what is supplied in house diet (~185 ppm iron). Of note, iron exposure of mice in our study was much shorter (4 weeks) than in human pregnancy (several months). Our second model, hepcidin KO mice, a genetic model of iron overload, is representative of iron overload caused by hepcidin deficiency in human hemochromatosis. Other differences between murine and human iron homeostasis include proportionally greater dietary iron absorption as well as iron losses in mice than in humans, but also greater resistance of mice to iron-induced tissue toxicity^54^. In our study, both genetic and dietary maternal iron loading resulted in higher placental iron and embryo iron stores. Although consequences of fetal iron excess in humans are not fully understood, it has been suggested that excessive iron supplementation of infants may have adverse effects on growth, risk of infections, cognition, and brain development^55^. In the hepcidin KO model, we did not detect any phenotypic effect of maternal genetic iron loading on embryo development, and hepcidin KO mice are viable, survive until adulthood, and are fertile. With dietary iron-loading, we observed a small but insignificant increase in embryo subcutaneous hemorrhaging. Given the resistance of mice to iron excess, additional studies are needed to better determine the long-term effects on offspring of excess iron during human pregnancy.

When both iron excess and inflammation were present in pregnant animals, we found an adverse synergy that resulted in dire consequences for the fetus. In our LPS model of acute inflammation, both dietary and genetic loading were associated with high incidence of embryo mortality or malformations. In our model of diet-induced obesity, representing a milder chronic inflammatory state, we similarly observed that iron loading caused embryo subcutaneous hemorrhaging or malformations. Our data question the safety of iron supplementation in women with acute or chronic inflammation.

Importantly, we defined the mechanism underlying the adverse synergy between acute inflammation and maternal iron excess. Our animal models show that maternal iron excess increased oxidative stress in the whole placenta and specifically in placental endothelial cells, which was further exacerbated in the presence of inflammation. Iron-induced oxidative stress sensitized placenta and embryo endothelium to inflammation-induced apoptosis, ultimately resulting in fetal death or malformations. Although also iron-dependent, this mechanism is distinct from ferroptosis as it does not involve iron-dependent changes in markers of lipid peroxidation, *Ptgs2*, or MDA. Of note, treating dams with vitamin E isomer α-Tocopherol prior to the inflammatory insult completely prevented endothelial apoptosis and fetal demise, likely due to the attenuation of placental inflammation. Vitamin E exerts its broad cytoprotective effects as an antioxidant by scavenging reactive oxygen species, inhibiting caspase activity^56,57^, and reducing inflammation^58–61^. Although the WHO reports no beneficial effect of routine vitamin E supplementation for women with or without a high risk pregnancy^62^, our study suggests that acute vitamin E therapy may be specifically beneficial in pregnancies with underlying inflammation when the women are receiving iron supplementation. We speculate that the mechanism of iron-dependent apoptosis and its prevention by vitamin E supplementation apply beyond pregnancy, to broader clinical problems associated with endothelial dysfunction, oxidative stress, and inflammation, and expect that iron loading could similarly worsen outcomes in hypertension, sickle cell disease, renal failure, and other cardiovascular and metabolic diseases.

We demonstrated that TNFα was the pathogenic factor synergizing with maternal iron excess. TNFα is a pleiotropic cytokine that regulates diverse biological functions including cell growth, viral replication, septic shock, inflammation, and autoimmunity^63^, and aberrant TNFα production is associated with many inflammatory diseases in humans. Although both our acute inflammatory LPS model and chronic inflammatory obesity model resulted in similar adverse embryo outcomes and placental apoptosis, there seem to be differences in how iron excess alters TNFα signaling in acute and chronic inflammatory settings. In acute inflammation caused by LPS injection, there was no difference in maternal TNFα production between iron-adequate and iron-loaded dams. Thus, iron excess in our acute inflammatory model alters downstream TNFα signaling rather than TNFα production. In contrast, in our obesity model, genetic iron loading increased circulating TNFα levels in the dam but not in placentas or embryos, suggesting that maternal iron excess did modulate TNFα production. TNFα is produced by adipose tissue and its secretion correlates with the degree of adiposity^64^, suggesting that iron excess may further promote TNFα production by adipose tissue in obesity.

Interestingly, embryopathies in obese pregnancies differed depending on the model of iron loading. Although we do not know the cause, it is likely that the extent and timing of maternal iron loading as well as the severity of their inflammation may affect their embryotoxicity. Hepcidin deficiency was associated with more severe maternal tissue and serum iron overload compared to dietary iron loading (although placental iron loading was similar between the two models). Hepcidin deficiency likely also promotes iron loading earlier during pregnancy compared to dietary iron loading, considering that maternal hepcidin suppression in WT mice only occurs in the second part of pregnancy^2^. Furthermore, hepcidin KO obese dams had higher levels of cytokines at the end of pregnancy compared to mothers with dietary iron loading. Thus, any of these factors could contribute to the specific embryo outcomes.

Notably, maternal anti-TNFα therapy was sufficient to prevent embryotoxicity in iron-loaded pregnancies in either the acute LPS inflammatory model, or in obese mice. The development of anti-TNFα therapy has been indispensable for the treatment of several inflammatory-mediated autoimmune conditions^65,66^. Monoclonal anti-TNFα antibodies are considered safe during pregnancy, although they are actively transported across the placenta particularly in the third trimester^67,68^, and are detectable in newborns until 6 months post-partum^67,69,70^. Despite measurable levels in offspring, studies report no association between anti-TNFα concentrations in infants at birth and risk of infection or in reaching developmental milestones in the first year of life^69,71^.

In summary, we investigated the consequences of excess maternal iron during healthy and inflamed pregnancy and discovered an adverse interaction. Maternal iron excess, accrued through dietary or genetic means, potentiated the adverse effects of inflammation during pregnancy, causing embryo malformations and demise. We observed this synergy between iron and acute inflammation utilizing an LPS-induced sepsis model and with chronic mild inflammation caused by diet-induced obesity. We provide specific evidence that the interaction between iron excess and inflammation is dependent on TNFα signaling causing lethal apoptotic damage to endothelial cells in the embryo and the placenta which can be prevented by maternal antioxidant therapy. Our data suggest that pregnant women could be at risk for adverse birth outcomes when the mother is exposed to both high iron levels and systemic inflammation during pregnancy and underscores the importance of screening pregnant women for iron deficiency before recommending iron supplementation. Given the increasing prevalence of inflammatory disorders worldwide, and that iron supplementation of pregnant women is universal, our study raises important questions about the safety of indiscriminate iron supplementation in inflamed pregnancy. Our work also details the mechanistic signature of this type of iron-dependent injury and potential therapies that could ameliorate it. Future studies will assess whether iron also represents a modifiable factor to decrease detrimental effects of inflammation in conditions other than pregnancy.

## Methods

### Animals

All animal experiments were approved by the University of California, Los Angeles (UCLA) Institutional Animal Care and Use Committee and were carried out in accordance with the Guide for Care and Use of Laboratory Animals (National Institutes of Health, Bethesda, MD). The following strains of animals were maintained on a 12-h light–dark schedule in a temperature- (22–25 °C) and humidity-controlled environment: wild-type (WT) C57BL/6J (Jackson Laboratory, Bar Harbor, ME, stock #000664), and hepcidin-1 knockout (KO) mice on C57BL/6 J background^72^. Unless specified, mice received a standard diet (PicoLab Rodent Diet 20, 5053 Irradiated, 185 ppm iron). For purified diets, iron content was determined by ICP-MS (Veterinary Diagnostic Laboratory, Michigan State University).

To model maternal dietary iron overload, female WT mice were fed a purified high iron diet containing 2500–5000 ppm carbonyl iron (Envigo Teklad TD. 160249 and TD.08043) for 1–3 weeks prior to mating and for the duration of pregnancy. Non-pregnant controls were subjected to the same iron treatment and were age-matched to pregnant dams.

For maternal obesity studies, female WT and hepcidin KO mice were fed a Western diet containing 100-ppm ferric citrate (Envigo Teklad TD.180722) starting at 3 weeks of age. After 8 weeks, WT mice were fed a Western diet supplemented with 3700-ppm carbonyl iron (Envigo Teklad TD.180723) for 1 week prior to mating and throughout pregnancy. Dietary iron loaded dams were compared to WT mice (iron adequate, fed standard diet) and hepcidin knockout dams (genetic iron overload) maintained on Western diet. The Western diet contains 0.2% total cholesterol, saturated fat >60% of total fat (42% calories from fat), and high sucrose (43% calories from carbohydrate), and 15% calories from protein.

After the indicated treatments, placentas and embryos were dissected from the maternal uterus and embryos were euthanized by decapitation. Placentas and embryo tissues were snap-frozen, or whole embryos and placentas were formalin-fixed and stored until analysis. Depending on the analysis, 1–3 placenta and embryo samples were randomly selected from each litter, so that all litters were represented in the analysis. Since samples were randomly selected, in assays where both placental and fetal tissues were analyzed, samples were generally not matched. We did not include in the analyses those embryos that were collected outside the uterus as a result of preterm deliveries.

Embryo “adverse outcomes” were scored based on whether abnormalities were present but not based on severity of abnormality. The score of 0 was assigned to embryos with no abnormalities, and the score of 100 to embryos with any abnormalities, and then the litter average of adverse outcomes was determined. The term “embryo loss” includes embryo resorption or fatal hemorrhaging, but not embryo malformations. “Embryo malformations” includes anencephaly, anophthalmia, hernia, and cleft palate. During the harvesting of embryos, embryos with anophthalmia and/or anencephaly were scored as positive for encephalic malformations, and negative for cleft palate or hernia. Thus, embryos with both anophthalmia and anencephaly (which were sometimes observed together) received the same score as embryos with either condition alone.

### Maternal systemic inflammation model

To induce inflammation in early gestation, pregnant mice on E8.5 were weighed and received a single subcutaneous injection of 0.5 µg/g LPS (Escherichia coli serotype O55:B5, Sigma-Aldrich L2880) or equivalent volume of sterile solvent as a control. Mice were weighed daily after injection, and mice that lost pregnancy weight gain were euthanized from E11.5-E18.5 to confirm embryo loss (miscarriage or resorption of embryos).

To induce inflammation in late gestation, pregnant mice on E15.5 received a single subcutaneous injection of LPS, 100 µg pam3csk4 (InvivoGen tlrl-pms), 30 µg flagellin (Bacillus subtilis, InvivoGen tlrl-bsfla), or single intravenous injection of 10 µg/g polyinosinic:polycytidylic acid (high molecular weight, InvivoGen tlrl-pic) or 10 µg/g lipotechoic acid (Staph aureus, InvivoGen tlrl-pslta). Preterm birth was recorded as the delivery of at least one pup within 24 h of injection. At the indicated times, mice were euthanized by isoflurane overdose and tissues collected for analysis.

For TNFα neutralizing experiments, pregnant E14.5 hepcidin KO dams received a single intravenous injection via the retroorbital sinus of 500 μg rat antibody targeting mouse TNFα (BioXCell) or rat IgG1 isotype control antibody targeting trinitrophenol (TNP, BioXCell) prior to LPS injection on E15.5 for 24 h. Pregnant hepcidin knockout dams fed a Western diet received intravenous injections of 250 μg of anti-TNFα or anti-TNP on E5.5, E8.5.5, E12.5, and E15.5, and mice were euthanized on E18.5. The antibodies are listed in Supplementary Table 2.

For antioxidant experiments, pregnant hepcidin KO dams received subcutaneous injections of 100 µg/g (±)-α-Tocopherol phosphate disodium salt (Sigma T2020) 14 and 2 h prior to LPS injection on E15.5 for 24 h.

### Cytokine and chemokine analysis

Cytokine and chemokine analyses were performed by the UCLA Integrated Molecular Technologies Core using a multiplex bead immunoassay (Millipore Milliplex Cytokine/Chemokine 32-plex Kit on Luminex FlexMap3D). Levels of mouse cytokines interleukin (IL)-1α, IL-1β, IL-2, IL-3, IL-4, IL-5, IL-6, IL-7, IL-9, IL-10, IL-12 (p40), IL-12 (p70), IL-13, IL-15, IL-17, leukemia inhibitory factor (LIF), interferon (IFN)-γ, and tumor necrosis factor (TNF)-α; chemokines interferon-inducible protein (IP)-10, keratinocyte derived chemokine (KC), LPS-induced CXC chemokine (LIX), monocyte chemotactic protein (MCP)-1, and Eotaxin/CCL11; colony-stimulating factors (CSF) macrophage (M)-CSF, monocyte induced by gamma-interferon (MIG), macrophage inflammatory protein (MIP)-1α, MIP-1β, MIP-2, regulated on activation normal T cells expressed and secreted (RANTES), granulocyte (G), granulocyte macrophage (GM)-CSF, and vascular endothelial growth factor (VEGF), were quantified in maternal serum 6 and 24 h after E15.5 LPS injection. For embryo serum, a separate cohort of dams were treated on E17.5 for 6 h and embryo serum from each litter was pooled to generate sufficient volume for analysis. For Western diet mouse experiments, cytokines in maternal serum collected on E18.5 were determined by the multiplex ELISA, and TNFα in pooled embryo serum and amniotic fluid was determined by a separate ELISA (Biolegend 430901) following the manufacturer’s instructions.

### Histopathology and immunohistochemistry

Placentas and whole embryos were fixed in 10% neutral buffered formalin for 48 h at room temperature, rinsed once in distilled water, and stored in 70% ethanol until processing. The tissues were paraffin-embedded and sectioned at 4 µm thickness by the UCLA Translational Pathology Core Laboratory. Prior to staining, sections were heated for 45 min at 50 °C and deparaffinized through serial changes of xylenes and ethanol and rehydrated to distilled water.

For TUNEL staining, tissue sections were processed using ApopTag Plus Peroxidase in situ Apoptosis Detection Kit (EMD Millipore #S7101) following the manufacturer’s instructions.

For immunohistochemistry, antigen retrieval was performed by boiling in Tris-EDTA (10 mM Tris base 1 mM disodium EDTA dihydrate, pH 9.0) for 10 min. Sections were quenched with BLOXALL (Vector Laboratories SP-6000) for 10 min and washed with TBS containing 0.1% Tween-20. Blocking was performed using 2.5% normal horse serum in PBS. Sections were incubated with primary antibodies in blocking buffer at 4 °C overnight in a humidified chamber. Negative control sections were incubated with normal IgG from the same species and at the same concentrations as primary antibodies. The secondary reaction was performed using ImmPRESS IgG (Vector Laboratories) conjugated to horseradish peroxidase (HRP) or alkaline peroxidase (AP). HRP and AP were developed using DAB and vector red per the manufacturer’s instructions (Vector Laboratories). Sections were counterstained with Gill’s hematoxylin. Light microscopy images were captured by a digital camera (Spot Imaging). The antibodies are listed in Supplementary Table 2.

### Cell culture

Primary human umbilical vein endothelial cells (HUVECs) pooled from 10 donors were obtained from ATCC (ATCC PCS-100-013) and cultured in complete endothelial cell growth medium (Cell Applications #211-500) at 37 °C in a 5% CO_2_ 95% air atmosphere. For experiments, HUVECs were seeded at 1 × 10^5^/well on collagen-coated plates (Corning BioCoat). All experiments were performed from passages 3 to 5.

For iron loading experiments, HUVECs were plated in regular media or media supplemented with 100 μM ferric ammonium citrate (FAC) (MP biomedicals #158040), or cupric chloride (Sigma #C-6917), or zinc sulfate (Fisher #Z-58) for 24 h. For iron depletion studies, HUVECs were treated with 100 µM FAC with and without 100 µM deferoxamine (Sigma D9533) for 24 h. The optimal time of culture was based on preliminary concentration and time-dependence studies.

For inflammation studies, HUVECs were treated with solvent, 2 mg/ml LPS O55:B5, or 50 ng/mL recombinant human IL6 (R&D Systems #206-IL), IFNγ (PeproTech #300-02), IL1α (PeproTech #200-01 A), IL1β (PeproTech #200-01B), or TNFα (Biolegend #570102) for the indicated time points. The optimal time of culture with cytokines was based on preliminary concentration and time-dependence studies.

For TNFα neutralizing experiments, HUVECs were treated with 100 µM FAC and 1 µg human TNFα neutralizing antibody (listed in Supplementary Table 2) for 24 h and stimulated with 50 ng/ml IL-1α, IL-1β, or TNFα for 16 h.

### RNA-Sequencing

RNA-Sequencing was performed by the UCLA Technology Center for Genomics & Bioinformatics on endothelial cells isolated from mouse placentas. Briefly, 5 placentas/dam were pooled, and endothelial cells were isolated by magnetic separation using the MidiMACS system following the manufacturer’s instructions (Miltenyi #130-095-927, #130-097-418). Total RNA was extracted using a RNeasy Micro kit (Qiagen) following the manufacturer’s instructions. Expression data were analyzed by comparing cells from the following groups of mice: iron adequate (WT) and iron loaded (HKO) pregnant mice injected on E15.5 with solvent or 0.5 µg/g LPS for 6 h (*n* = 4 dams/group). Data were sequenced on Illumina HiSeq 3000 for a single-read 50 bp run, and depth of coverage was 2 lanes for >30 million reads/sample. Partek flow and Ingenuity Pathway Analysis (IPA) were used for bioinformatics methods and data analysis, respectively. The reads were mapped to the latest UCSC transcript set using STAR-2.6.1d. The Partek E/M was used to quantify reads to an annotation model. After obtaining gene counts, the counts were normalized by TPM. The Principal Component Analysis was applied to the transcript counts. The differential gene expressions were examined using DESeq2 algorithm. Canonical pathway analysis was performed by IPA using significantly differentially expressed genes (genes filtered by *P* < 0.05).

### Non-heme iron measurements

Serum was obtained from maternal and embryo blood by centrifugation at 2700 × *g* for 10 min. Serum iron concentration was measured by colorimetric spectrophotometry using an iron calibrator (Iron-SL, Sekisui Diagnostics 157-30)^73^, excluding hemolyzed samples. For maternal liver iron measurements, 75 μL of whole liver homogenate was weighed and incubated in 1125 μL protein precipitation solution (0.53 N HCl 5.3% trichloroacetic acid) at 100 °C for 1 h. For embryo tissue iron measurements, half of each placenta and liver were weighed and homogenized in 1200 μL protein precipitation solution prior to boiling. Samples were centrifuged at 17,000 × *g* for 10 min and the supernatant was analyzed by colorimetric spectrophotometry using an iron standard (1000-ppm iron in 3% HCl, Ricca Chemical Company).

### Serum endotoxin assay

Endotoxin in maternal and embryo serum was determined by a limulus amebocyte assay kit following the manufacturer’s instructions (BioWhittaker #50-648U).

### Serum progesterone assay

Progesterone in maternal serum was measured by ELISA according to the manufacturer’s instructions (Cayman Chemicals #582601).

### Lipid peroxidation assay

Lipid peroxidation was quantified in serum and embryonic tissues by colorimetric spectrophotometry using the Thiobarbituric Acid Reactive Substances (TCA Method) Assay Kit (Cayman Chemicals #700870) following the manufacturer’s instructions.

### Western blot analysis

Placenta pieces from pregnant mice treated with LPS or solvent on E15.5 were homogenized in RIPA lysis buffer with freshly added protease inhibitor cocktail (Santa Cruz #24948). For cell culture experiments, HUVECs were lysed in RIPA buffer by mechanical disruption. Tissue and cell lysates were centrifuged at 17,000 × *g* for 15 min at 4 °C and protein concentration was measured by the bicinchoninic acid assay using bovine serum albumin as a standard. Proteins from placentas (30 μg/lane) and HUVECs (20 μg/lane) were separated by SDS-PAGE and transferred to nitrocellulose membranes. Membranes were blocked for 1 h in 5% w/v dried nonfat milk or bovine serum albumin in TBS with 0.1% Tween-20 and incubated with primary antibodies in blocking buffer overnight at 4 °C The secondary reaction was performed using HRP-conjugated anti-rabbit IgG diluted in blocking buffer. Protein blots were visualized by chemiluminescence using the ChemiDoc XRS+ imaging system and quantified using Image Lab software (Bio-RAD). The antibodies, dilutions, and catalog numbers are listed in Supplementary Table 2.

### Gene expression quantification by RT-PCR

Frozen mouse tissues or cell cultures were homogenized in TRIzol Reagent (Life Technologies) and total RNA was isolated by chloroform extraction. One μg (mouse tissues) or 500 ng (cell cultures) of RNA was reverse transcribed using the iScript cDNA Synthesis Kit (Bio-RAD). Quantitative real-time PCR was performed on cDNA using SsoAdvanced SYBR Green Supermix (Bio-RAD) on the CFX Real-Time PCR Detection System (Bio-RAD). Samples were measured in duplicate and target genes were normalized to *Rpl4 or Hprt*. Data are expressed as 2^−∆∆Ct^. The primers are listed in Supplementary Table 3.

### Statistical analysis

Statistical analysis was performed using GraphPad Prism 9. Data are presented as individual values representing the mean ± SEM. Statistical differences between groups were determined by one-way ANOVA followed by Holm-Sidak for multiple comparisons for normally distributed values, one-way ANOVA on ranks followed by Dunn’s method for multiple comparisons of non-normally distributed values, two-tailed student’s *t*-test for normally distributed values, Mann–Whitney *U* test for non-normally distributed values, or Fisher’s exact test. Shapiro-Wilk test was used to test normality. Statistical test, number of animals in each group, experimental replicates, and *P*-value are indicated in each figure panel or legend. A *P*-value of <0.05 was considered significant.

### Reporting summary

Further information on research design is available in the Nature Research Reporting Summary linked to this article.

## Supplementary information

Supplementary Information

Reporting Summary

## Data Availability

All data are included in this published article and its Supplementary Information files. The RNA-Seq data that support the findings of this study have been deposited in the NCBI Gene Expression Omnibus^74^ and are accessible through the GEO Series accession number GSE153528. Source data are provided with this paper.

## Source data

Source Data
