# Supplementary material for: Iron-dependent apoptosis causes embryotoxicity in inflamed and obese pregnancy
Source: Nat Commun. 2021 Jun 29;12:4026. doi: 10.1038/s41467-021-24333-z (PMC8242011; doi:10.1038/s41467-021-24333-z)
Supplement: Supplementary file 1 — Supplementary Information [file 41467_2021_24333_MOESM1_ESM.pdf]

SUPPLEMENTARY INFORMATION

Iron-dependent apoptosis causes embryotoxicity in inflamed and obese pregnancy

Allison L Fisher, Veena Sangkhae, Kamila Balušíková, Nicolaos J Palaskas, Tomas Ganz, Elizabeta Nemeth

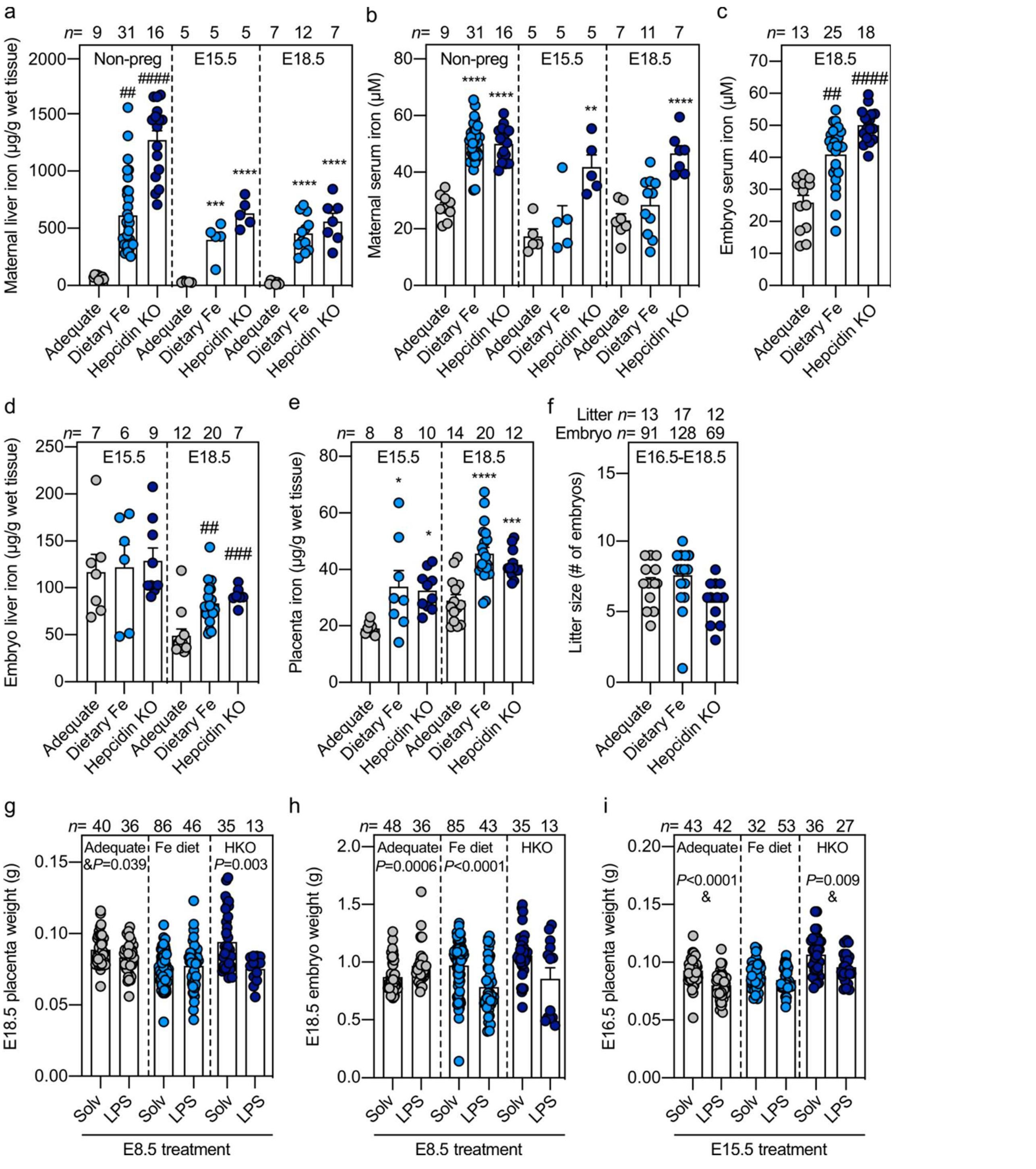

Supplementary Figure 1. Maternal, placental, and embryo iron status in dietary and genetic iron overload during pregnancy. Iron-adequate mice were maintained on standard chow (185 ppm iron). Dietary iron-loaded dams were fed a high iron diet (2,500-5,000 ppm iron) for 1-3 weeks prior to mating and during pregnancy. Genetic iron-loading model were hepcidin-1 KO dams fed standard chow. Non-pregnant females received the same iron treatment and were age-matched to pregnant dams. Pregnant iron-loaded dams were analyzed at E15.5 and E18.5 and compared to iron-adequate controls. **(a-b)** Maternal non-heme iron measurements in liver and serum. **(c-d)** Embryo non-heme iron measurements in serum and liver from randomly selected embryos. Embryo serum was not analyzed at E15.5 due to insufficient volume for analysis. **(e)** Placenta non-heme iron concentrations from randomly selected placentas. **(f)** Litter size from iron-adequate and iron-loaded dams (# of embryos counted in E16.5-E18.5 pregnancies). **(g-i)** Maternal systemic inflammation was induced by a single subcutaneous injection of 0.5 µg/g LPS in the interscapular area either on E8.5 or E15.5. Weights of E18.5 **(g)** placentas or **(h)** embryos after maternal LPS treatment on E8.5. **(i)** E16.5 placenta weights after maternal E15.5

LPS treatment. **(g-i)** Embryo weights represent healthy and malformed embryos but exclude resorbing embryos in which weights were not accurately obtained. Error bars represent mean ± s.e.m. Statistical differences between groups were determined by two-tailed Mann-Whitney *U*, two-tailed Student's *t*-test (denoted by &), one-way ANOVA for normally distributed values followed by Holm-Sidak method for multiple comparisons versus iron-adequate group (denoted by *P<0.05, **P<0.01, ***P<0.001, ****P<0.0001), or one-way ANOVA on ranks followed by Dunn's method for multiple comparisons versus iron-adequate group (denoted by #P<0.05, ##P<0.01, ###P<0.001, ####P<0.0001). Animal numbers and *P*-values are indicated in each panel. Source data are provided as a Source Data file.

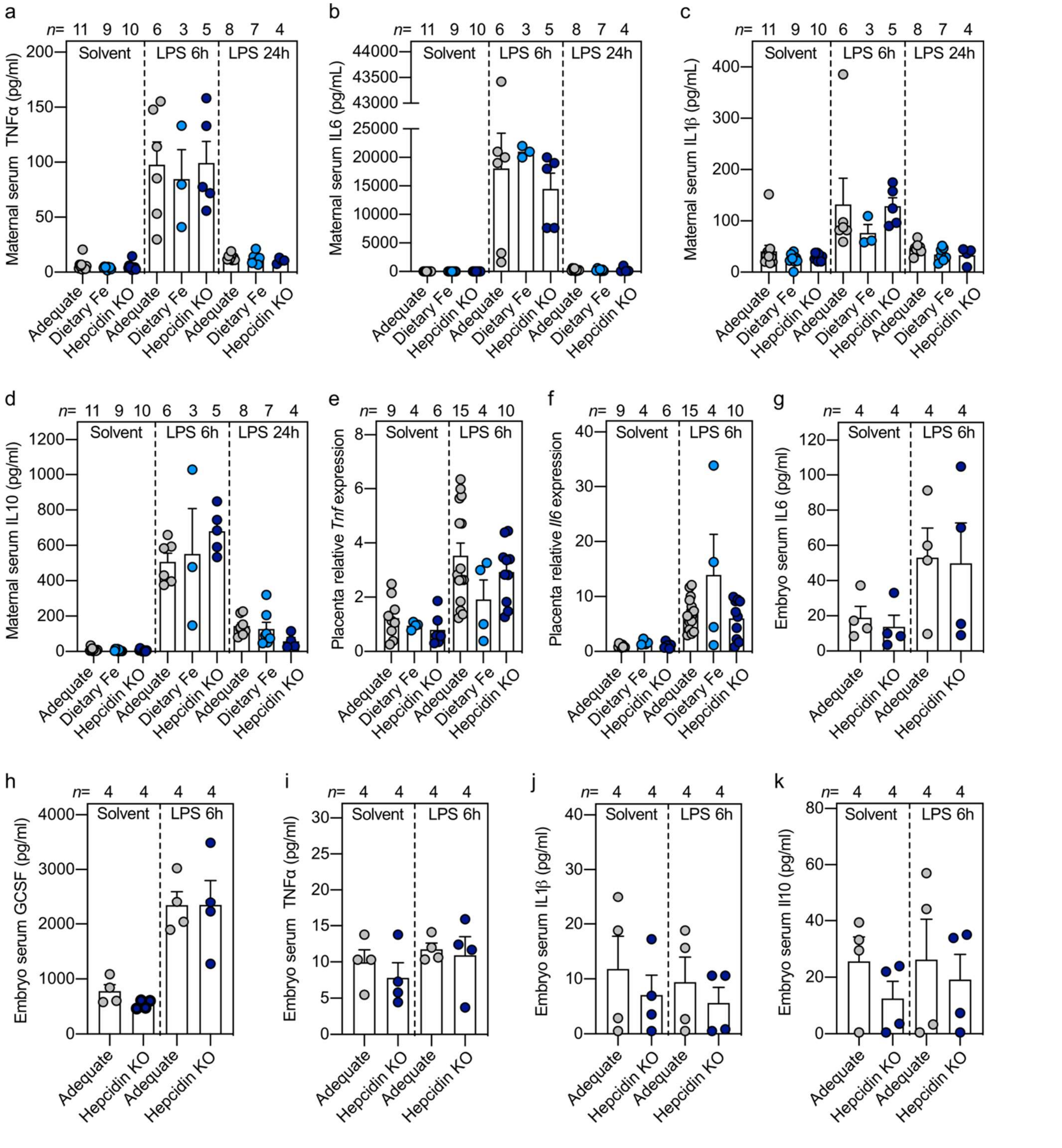

Supplementary Figure 2. Maternal, placental, and embryo inflammation in dietary and genetic iron overload during pregnancy. Iron-adequate mice were maintained on standard chow (185 ppm iron). Dietary iron-loaded dams were fed a high iron diet (2,500-5,000 ppm iron) for 1-3 weeks prior to mating and during pregnancy. Genetic iron-loading model were hepcidin-1 KO dams fed standard chow. **(a-d)** Cytokines TNFα, IL6, IL1β, and IL10 in maternal serum 6 and 24 hours after maternal LPS treatment on E15.5. **(e-f)** Placenta mRNA expression of *Tnf* and *Il6* normalized to *Rpl4* 6 hours after maternal LPS injection on E15.5. **(g-k)** Embryo serum IL6, GCSF, TNFα, IL1β, and IL10 6 h after maternal LPS treatment on E17.5, in a separate cohort where embryo blood was pooled from each litter to obtain sufficient serum for analysis. Error bars represent mean ± s.e.m. Animal numbers are indicated in each panel. Source data are provided as a Source Data file.

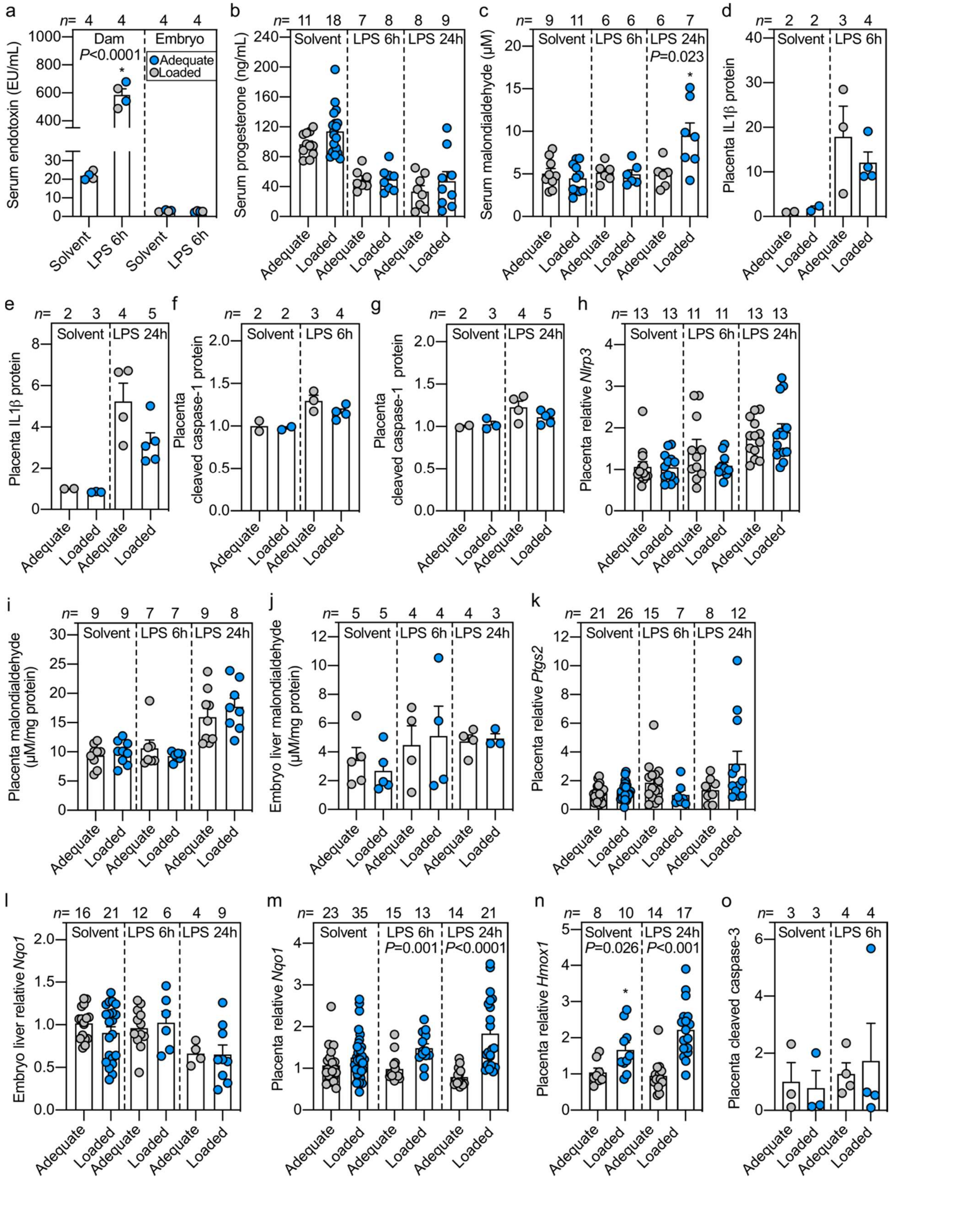

Supplementary Figure 3. Comparison of cell death markers and mediators induced by LPS in placentas and embryos from iron-adequate and iron-loaded pregnancies. (a) Endotoxin in maternal serum and in pooled embryo serum after maternal subcutaneous LPS injection (0.5 μg/g) on E17.5 for 6 hours. **(b-n)** Iron-adequate and iron-loaded dams were treated with subcutaneous LPS (0.5 μg/g) on E15.5 for 6 and 24 hours. **(b)** Maternal serum progesterone and **(c)** serum malondialdehyde. **(d-g)** Quantification of IL-1β and cleaved

caspase-1 by Western blot in whole placenta lysates. **(h)** Placental *Nlrp3* mRNA expression. **(i-j)** Malondialdehyde measurements in placenta and embryo livers. **(k)** Placental *Ptgs2* mRNA expression. **(l-m)** Embryo liver and placenta *Nqo1* mRNA expression. **(n)** Placenta *Hmox1* mRNA expression. **(o)** Quantification of cleaved caspase-3 by Western blot in whole placenta lysates after maternal LPS injection for 6 hours. **(d-o)** Embryo and placenta samples were randomly selected for analysis. Error bars represent mean ± s.e.m. Statistical differences between groups were determined by two-tailed Mann-Whitney *U* for non-normally distributed values or two-tailed Student's *t*-test for normally distributed values (denoted by *). *P* values and animal numbers are indicated in each panel. Source data are provided as a Source Data file.

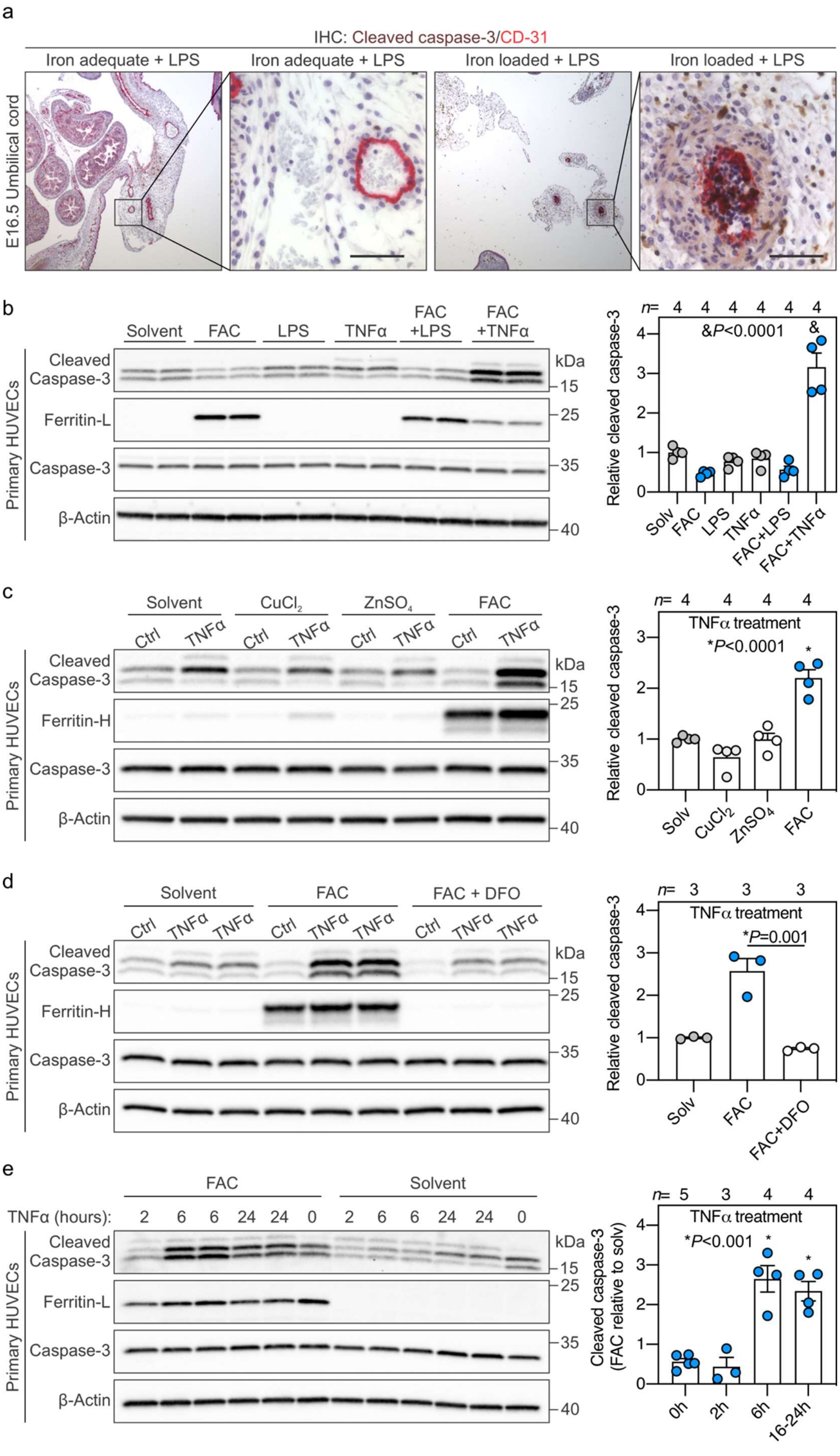
a
IHC: Cleaved caspase-3/CD-31
Iron adequate + LPS
Iron adequate + LPS
Iron loaded + LPS
Iron loaded + LPS
E16.5 Umbilical cord
b
Solvent
FAC
LPS
TNFα
FAC +LPS
FAC +TNFα
Primary HUVECs
Cleaved Caspase-3
Ferritin-L
Caspase-3
β-Actin
kDa
15
25
35
40
n= 4 4 4 4 4 4
&P<0.0001
&
Relative cleaved caspase-3
Solv
FAC
LPS
TNFα
FAC+LPS
FAC+TNFα
c
Solvent
CuCl2
ZnSO4
FAC
Ctrl
TNFα
Cleaved Caspase-3
Ferritin-H
Caspase-3
β-Actin
n= 4 4 4 4
TNFα treatment
*P<0.0001
Relative cleaved caspase-3
Solv
CuCl2
ZnSO4
FAC
d
Solvent
FAC
FAC + DFO
Ctrl
TNFα
TNFα
Cleaved Caspase-3
Ferritin-H
Caspase-3
β-Actin
n= 3 3 3
TNFα treatment
*P=0.001
Relative cleaved caspase-3
Solv
FAC
FAC+DFO
e
FAC
Solvent
TNFα (hours): 2 6 6 24 24 0 2 6 6 24 24 0
Cleaved Caspase-3
Ferritin-L
Caspase-3
β-Actin
n= 5 3 4 4
TNFα treatment
*P<0.001
Cleaved caspase-3 (FAC relative to solv)
0h
2h
6h
16-24h

Supplementary Figure 4. Potentiation of TNFα-induced apoptosis is specific to iron. (a) Immunohistochemistry of paraffin-embedded mouse umbilical cords for cleaved caspase-3 (brown) and CD31 (red) after iron-adequate and iron-loaded dams were treated with LPS on E15.5 for 24 hours (*n*=1 section/group). Scale bar= 50 μm. **(b)** HUVECs were treated with solvent or 100 μM ferric ammonium citrate (FAC) for 24 h and simulated with 2 mg/ml LPS or 50 ng/ml TNFα for 16 hours. Western blot (left) and quantitation (right) of cleaved caspase-3 normalized to β-actin (*n*=4 independent experiments). **(c)** HUVECs were treated with solvent, 100 μM FAC, 100 μM copper chloride ($CuCl_2$), or 100 μM zinc sulfate ($ZnSO_4$) and stimulated with 50 ng/ml TNFα for 16 hours. Western blot for cleaved caspase-3 (left) and quantitation of *n*=4 independent experiments (right). β-actin was used as a loading control. **(d)** HUVECs were treated with 100 μM FAC with and without 100 μM iron chelator deferoxamine for 24 hours prior to stimulation with 50 ng/ml TNFα for 16 hours. Western blot for cleaved caspase-3 (left) and quantitation of *n*=3 independent experiments (right). β-actin was used as a loading control. **(e)** HUVECs were treated with solvent or 100 μM ferric ammonium citrate (FAC) for 24 hours and stimulated with 50 ng/ml TNFα for 0 (*n*=5) 2 (*n*=3), 6 (*n*=4) and 24 hours (*n*=4). Western blot (left) and quantitation (right) of cleaved caspase-3 normalized to β-actin (*n*=3-5 replicates). **(b-e)** Error bars represent mean ± s.e.m. Statistical differences between groups were determined by two-way ANOVA (denoted by &) or one-way ANOVA for normally distributed values with Holm-Sidak method for multiple comparisons versus solvent group (denoted by *). *P* values and experimental replicates are indicated in each panel. Source data are provided as a Source Data file.

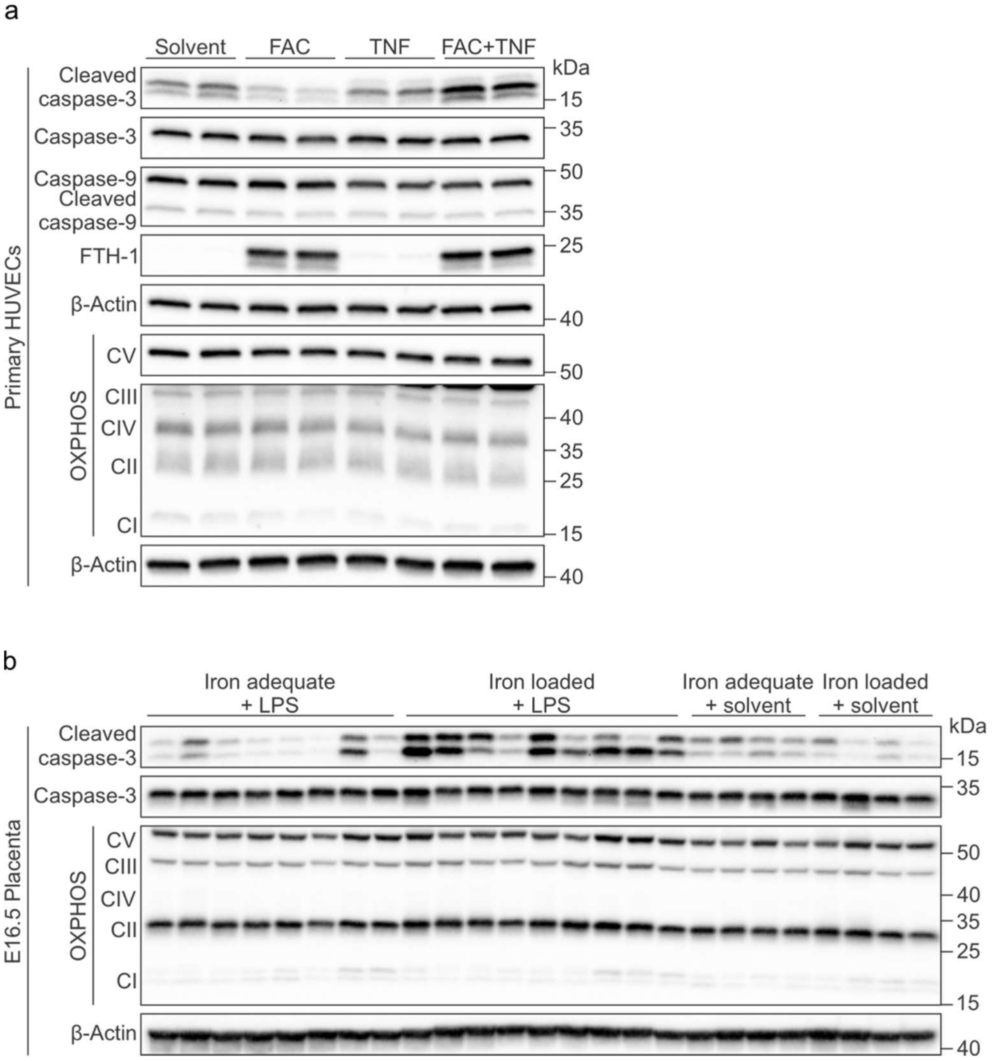

Supplementary Figure 5. Comparison of the intrinsic and extrinsic pathways in iron-potentiated apoptosis. (a) Primary HUVECs were treated with solvent or 100 μM FAC for 24 hours prior to stimulation with 50 ng/ml TNFα for 16 hours. Western blot for cleaved caspase-3, -9, and mitochondrial complexes CI-CV (OXPHOS). Ferritin heavy chain (FTH1) is a marker of cellular iron loading. Representative image of *n*=3 independent experiments. **(b)** Iron-adequate and iron-loaded dams were treated with solvent (both *n*=4) or LPS (iron adequate *n*=8, iron loaded *n*=9) on E15.5 for 24 hours. Placentas were randomly selected for analysis. Western blot for cleaved caspase-3 and mitochondrial OXPHOS complexes CI-CV. β-actin was used as a loading control. Source data are provided as a Source Data file.

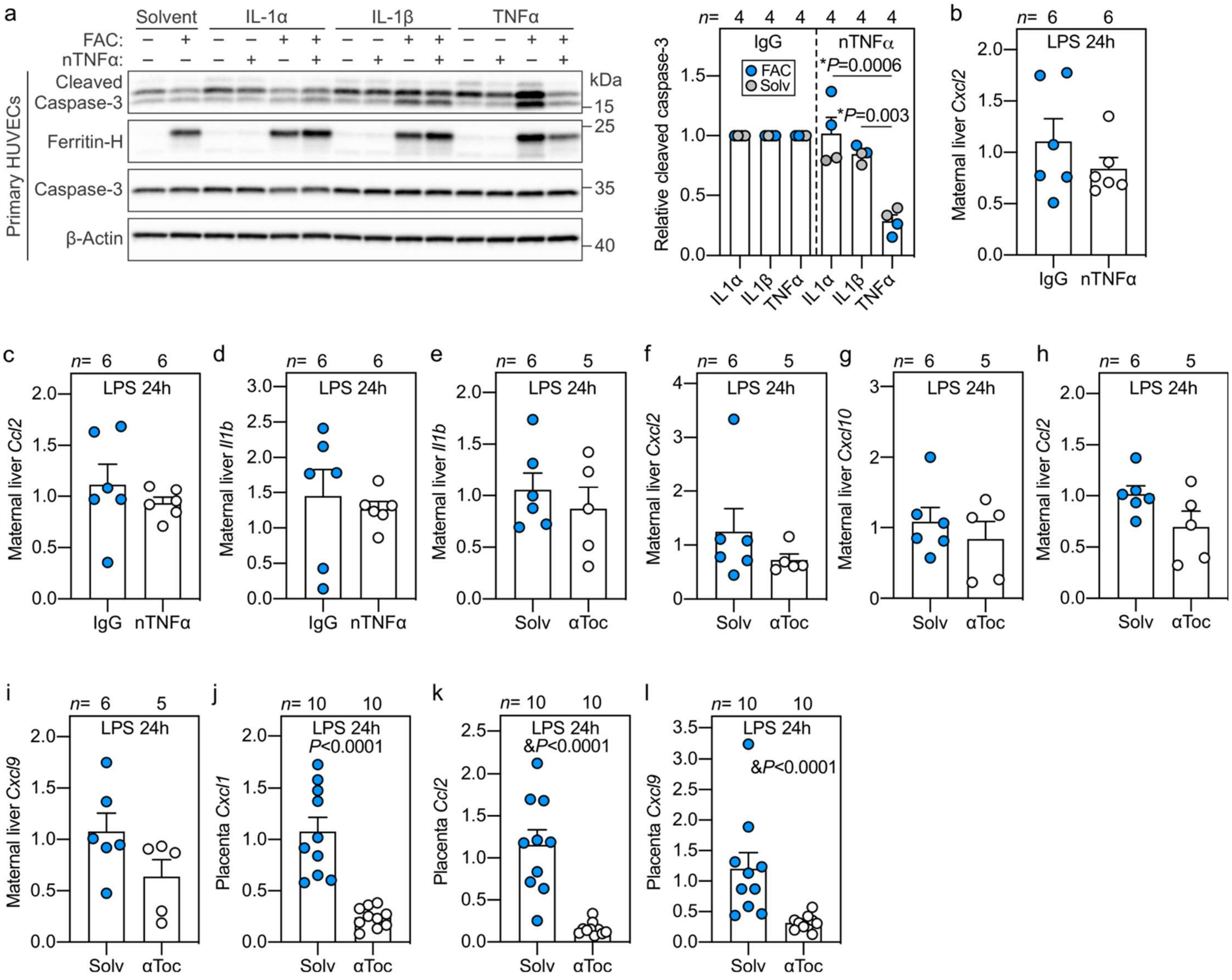

Supplementary Figure 6. Maternal pretreatment with neutralizing TNFα antibody or αTocopherol does not attenuate LPS-induced maternal inflammation. (a) The specificity of TNFα neutralizing antibody was tested in control and 100 μM ferric ammonium citrate (FAC)-loaded HUVECs treated with 50 ng/ml of IL-1α, IL-1β, or TNFα with and without 1 μg neutralizing TNFα antibody (nTNFα). Western blot for cleaved caspase-3 (left) and quantitation of *n*=4 independent experiments. β-actin was used as a loading control. **(b-d)** Hepcidin KO dams were treated intravenously via the retroorbital sinus with 500 μg TNFα neutralizing antibody (nTNF) or isotype control IgG antibody targeting trinitrophenol on E14.5, 15 hours prior to subcutaneous LPS injection (0.5 μg/g) on E15.5 for 24 hours. Inflammatory genes were measured by qRT-PCR in maternal liver: **(b)** *Cxcl2*, **(c)** *Ccl2*, and **(d)** *Il1b*. **(e-l)** Hepcidin KO dams were treated with subcutaneous αTocopherol (vitamin E, 100 ug/g) 14 and 2 hours prior to LPS injection on E15.5 for 24 hours. Inflammatory genes in maternal liver: **(e)** *Il1b*, **(f)** *Cxcl2*, **(g)** *Cxcl10*, **(h)** *Ccl2*, and **(i)** *Cxcl9*; **(j-l)** placental mRNA expression of *Cxcl1*, *Ccl2*, and *Cxcl9*. Placenta samples were randomly selected from each litter. Error bars represent mean ± s.e.m. **(a-l)**. Differences between groups was determined by two-tailed Student's *t*-test for normally distributed values, two-tailed Mann-Whitney *U* for non-normally distributed values (denoted by &), or one-way ANOVA for normally distributed values followed by Holm-Sidak method for multiple comparisons versus control group (denoted by *). *P* values, experimental replicates, and animal numbers are indicated in each panel. Source data are provided as a Source Data file.

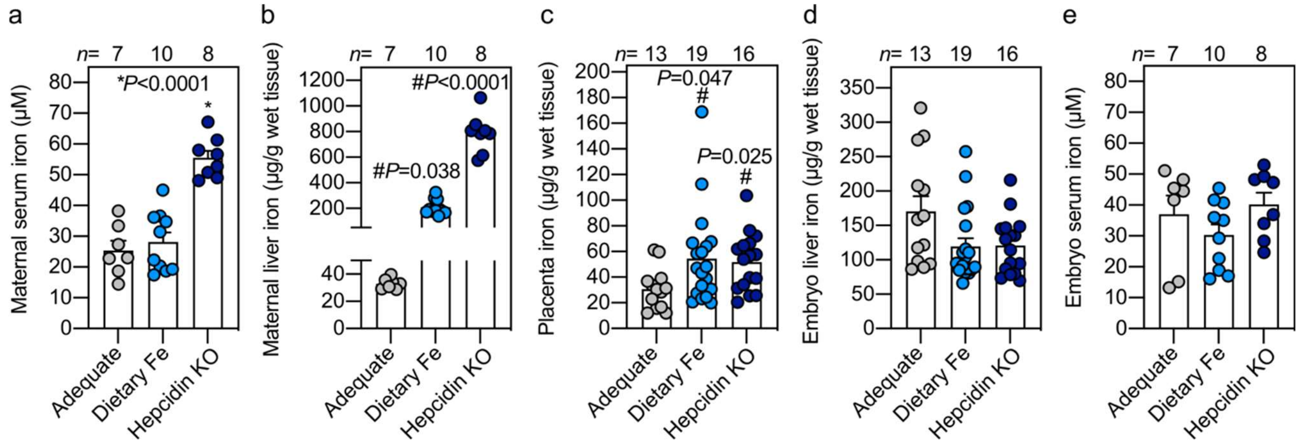

Supplementary Figure 7. Iron parameters in iron-adequate, dietary iron supplemented, and hepcidin KO obese mice. To induce obesity, wild-type C57BL/6 or hepcidin KO mice were fed a Western diet (100 ppm iron) starting at 3 weeks of age. Dietary iron-loaded dams were fed a Western diet supplemented with 3,700 ppm carbonyl iron for 1 week prior to mating and for the duration of pregnancy. Iron-loaded dams were analyzed at E18.5 and compared to iron-adequate controls. **(a)** Maternal serum iron and **(b)** liver iron concentrations. Iron measurements at E18.5: **(c)** placenta, **(d)** embryo liver iron, and **(e)** embryo serum iron. Embryo and placenta samples were randomly selected from each litter. Error bars represent mean ± s.e.m. Statistical differences between groups were determined by one-way ANOVA for normally distributed values followed by Holm-Sidak method for multiple comparisons versus adequate group (denoted by *) or one-way ANOVA on ranks followed by Dunn's method for multiple comparisons versus adequate group (denoted by #). *P* values and animal numbers are indicated in each panel. Source data are provided as a Source Data file.

Supplementary Table 1. Interaction of maternal iron excess and PAMPs in pregnancy										
PAMP	Mimetic	TLR	Dose/ Route	GD/ time	Iron adequate			Iron loaded		
					N	*Saa1* fold-change (Mean±SD)	Embryo outcome	N	*Saa1* fold-change (Mean±SD)	Embryo outcome
LPS *E. coli* O55:B5	Gram-negative bacteria	TLR4	0.5 µg/g SQ	E15.5, 24 hours	8	251 ± 92	Normal	4	284 ± 42	Adverse
Poly I:C	dsDNA virus	TLR3	10 µg/g IV	E15.5, 24 hours	3	289 ± 123	Normal	5	197 ± 38	Normal
LTA (*S. aureus*)	Gram-positive bacteria	TLR2	20 µg/g IV	E15.5, 24 hours	3	227 ± 97	Normal	4	139 ± 33	Normal
Pam3csk4	Triacylated lipopeptide	TLR1/2	100 µg SQ	E15.5, 24 hours	3	229 ± 72	Normal	4	330 ± 167	Normal
Flagellin (*B. subtilis*)	Flagellin	TLR5	30 µg SQ	E15.5, 24 hours	3	76 ± 46	Normal	3	33 ± 19	Normal

Supplementary Table 1. Interaction of maternal iron excess and PAMPs in pregnancy. Pregnant iron-adequate WT dams and iron-loaded hepcidin KO dams were treated with the indicated PAMPs on E15.5 to evaluate the interaction of maternal iron excess with inflammatory pathways triggered by a broader spectrum of pathogen-derived molecules from viruses and bacteria. PAMP= pathogen associated molecular patter, LPS= lipopolysaccharide, Poly I:C= polyinosinic:polycytidylic acid, LTA= lipoteichoic acid. TLR= toll like receptor. SQ= subcutaneous, IV=intravenous, GD= gestational day, Saa1= serum amyloid A1. Source data are provided as a Source Data file.

Supplementary Table 2. Antibodies	
**Target protein**	**Primary antibody**
Mouse and human cleaved caspase-3	Rabbit monoclonal antibody 9664, Cell Signaling Technology, 1:1,000
Mouse and human total caspase-3	Rabbit polyclonal antibody 9662, Cell Signaling Technology, 1:5,000
Mouse and human ferritin heavy chain	Rabbit monoclonal antibody 4393, Cell Signaling Technology, 1:25,000
Mouse and human ferritin light chain	Goat polyclonal NBP1-06986, Novus Biologicals, 1:5,000
Mouse and human β-actin	Monoclonal antibody-peroxidase A3854, Sigma, 1:50,000
Mouse IL-1 beta /IL-1F2	Goat polyclonal antibody AF401, R&D Systems, 1:1,000
Mouse cleaved caspase-1	Rabbit monoclonal antibody 89332, Cell Signaling Technology, 1:3,000
Human caspase-9	Mouse monoclonal antibody 9508, Cell Signaling Technology. 1:1,000
Mouse and human electron transport chain	Total OXPHOS Rodent WB Antibody Cocktail ab110413, Abcam, 1:5,000
Mouse CD31/PECAM-1	Goat polyclonal antibody AF3628, Novus Biologicals, 1:1,000
**Secondary antibodies:**	
Anti-mouse IgG HRP antibody 7076, Cell Signaling Technology, 1:5,000	
Anti-rabbit IgG HRP antibody 7074, Cell Signaling Technology, 1:5,000	
Anti-goat IgG HRP antibody 2354, Santa Cruz, 1:5,000	
ImmPRESS horse anti-rabbit IgG HRP detection kit MP-7401, Vector Laboratories	
ImmPRESS horse anti-goat IgG AP detection kit MP-5405, Vector Laboratories	
**Neutralizing antibodies:**	
Human TNFα neutralizing	Rabbit monoclonal antibody 7321, Cell Signaling Technology
Mouse TNFα neutralizing	Rat monoclonal antibody clone XT3.11, BioXcell
Rat IgG isotype control, anti-trinitrophenol	Rat monoclonal antibody clone TNP6A7, BioXcell

Supplementary Table 2. Antibodies.

Supplementary Table 3. List of qPCR primers.	
**Gene**	**Sequence**
Mouse *Tnf*	Forward: 5’- AAT GGC CTC CCT CTC ATC AG -3’<br>Reverse: 5’- GCT ACG ACG TGG GCT ACA GG -3’
Mouse *Il6*	Forward: 5’- CTC TGC AAG AGA CTT CCA TCC AGT -3’<br>Reverse: 5’- CGT GGT TGT CAC CAG CAT CA -3’
Mouse *Cxcl9*	Forward: 5’- GCC ATG AAG TCC GCT GTT CT -3’<br>Reverse: 5’- TAG GGT TCC TCG AAC TCC ACA -3’
Mouse *Il1b*	Forward: 5’- AAG GAG AAC CAA GCA ACG ACA AAA -3’<br>Reverse: 5’- TGG GGA ACT CTG CAG ACT CAA ACT -3’
Mouse *Nqo1*	Forward: 5’- CAC GGG GAC ATG AAC GTC AT -3’<br>Reverse: 5’- GGA GTG TGG CCA ATG CTG TA -3’
Mouse *Nlrp3*	Forward: 5’- AAA ATG CCT TGG GAG ACT CA -3’<br>Reverse: 5’- AAG TAA GGC CGG AAT TCA CC -3’
Mouse *Ptgs2*	Forward: 5’- TGA GTA CCG CAA ACG CTT CT -3’<br>Reverse: 5’- CAG CCA TTT CCT TCT CTC CTG T -3’
Mouse *Saa1*	Forward: 5’- AGT CTG GGC TGC TGA GAA AA -3’<br>Reverse: 5’- ATG TCT GTT GGC TTC CTG GT -3’
Mouse *Ccl2*	Forward: 5’- AGG TCC CTG TCA TGC TCC TG -3’<br>Reverse: 5’- TCA TTG GGA TCA TCT TGC TG -3’
Mouse *Cxcl2*	Forward: 5’- GAA GTC ATA GCC ACT CTC AAG G -3’<br>Reverse: 5’- CCT CCT TTC CAG GTC AGT TAG C -3’
Mouse *Cxcl10*	Forward: 5’- CCA CGT GTT GAG ATC ATT GCC -3’<br>Reverse: 5’- TCA CTC CAG TTA AGG AGC CC -3’
Mouse *Cxcl1*	Forward: 5’- AGA CCA TGG CTG GGA TTC AC -3’<br>Reverse: 5’- AGT GTG GCT ATG ACT TCG GT -3’
Mouse *Cd31*	Forward: 5’- CAG GAC CAC GTG TTA GTG TT -3’<br>Reverse: 5’- ACT CCT GAT GGG TTC TGA CT -3’
Mouse *Tfrc*	Forward: 5’- TCA TGA GGG AAA TCA ATG AT -3’<br>Reverse: 5’- GCC CCA GAA GAT ATG TCG GAA -3’
Mouse *Hmox1*	Forward: 5’- CCT CAC AGA TGG CGT CAC TT -3’<br>Reverse: 5’- TCT GCA GGG GCA GTA TCT TG -3’
Mouse *Hprt*	Forward: 5’- CTG GTT AAG CAG TAC AGC CCC AA -3’<br>Reverse: 5’- CAG GAG GTC CTT TTC ACC AGC -3’
Mouse *Rpl4*	Forward: 5’- TGA AAA GCC CAG AAA TCC AA -3’<br>Reverse: 5’- AGT CTT GGC GTA AGG GTT CA -3’
Human *HPRT*	Forward: 5’- GCC CTG GCG TCG TG ATTA GT -3’<br>Reverse: 5’- AGC AAG ACG TTC AGT CCT GTC -3’
Human *NQO1*	Forward: 5’- GCT GGT TTG AGC GAG TGT TC -3’<br>Reverse: 5’- CTG CCT TCT TAC TCC GGA AGG -3’
Human *HMOX1*	Forward: 5’- ACC TTC CCC AAC ATT GCC AG -3’<br>Reverse: 5’- CAA CTC CTC AAA GAG CTG GAT -3’
Human *TFRC*	Forward: 5’- AGT TGA ACA AAG TGG CAC GAG -3’<br>Reverse: 5’- AGC AGT TGG CTG TTG TAC CTC -3’

Supplementary Table 3. List of qPCR primers.
