# Supplementary material for: Iron-dependent apoptosis causes embryotoxicity in inflamed and obese pregnancy
Source: Nat Commun. 2021 Jun 29;12:4026. doi: 10.1038/s41467-021-24333-z (PMC8242011; doi:10.1038/s41467-021-24333-z)
Supplement: Supplementary file 2 — Reporting Summary [file 41467_2021_24333_MOESM2_ESM.pdf]

nature research

Corresponding author(s): Elizabeta Nemeth

Last updated by author(s): May 1, 2021

Reporting Summary

Nature Research wishes to improve the reproducibility of the work that we publish. This form provides structure for consistency and transparency in reporting. For further information on Nature Research policies, see our Editorial Policies and the Editorial Policy Checklist.

Statistics

For all statistical analyses, confirm that the following items are present in the figure legend, table legend, main text, or Methods section.

n/a	Confirmed	
☐	☒	The exact sample size (*n*) for each experimental group/condition, given as a discrete number and unit of measurement
☒	☐	A statement on whether measurements were taken from distinct samples or whether the same sample was measured repeatedly
☐	☒	The statistical test(s) used AND whether they are one- or two-sided<br>*Only common tests should be described solely by name; describe more complex techniques in the Methods section.*
☒	☐	A description of all covariates tested
☐	☒	A description of any assumptions or corrections, such as tests of normality and adjustment for multiple comparisons
☐	☒	A full description of the statistical parameters including central tendency (e.g. means) or other basic estimates (e.g. regression coefficient) AND variation (e.g. standard deviation) or associated estimates of uncertainty (e.g. confidence intervals)
☐	☒	For null hypothesis testing, the test statistic (e.g. *F*, *t*, *r*) with confidence intervals, effect sizes, degrees of freedom and *P* value noted<br>*Give P values as exact values whenever suitable.*
☒	☐	For Bayesian analysis, information on the choice of priors and Markov chain Monte Carlo settings
☒	☐	For hierarchical and complex designs, identification of the appropriate level for tests and full reporting of outcomes
☒	☐	Estimates of effect sizes (e.g. Cohen's *d*, Pearson's *r*), indicating how they were calculated

Our web collection on statistics for biologists contains articles on many of the points above.

Software and code

Policy information about availability of computer code

Data collection	Light microscopy images were captured by a digital camera (Spot Imaging).<br>Protein blots were visualized by chemiluminescence using the ChemiDoc XRS+ imaging system and quantified using Image Lab software (Bio-RAD, version 6.1.0).<br>Quantitative real-time PCR was performed on cDNA using SsoAdvanced SYBR GREEN supermix (Bio-RAD) on the CFX Real-Time PCR Detection System (Bio-RAD).
Data analysis	Statistical Analysis was done using Prism 9 (GraphPad).<br>Details on the RNA-Seq analysis methods done by the UCLA Technology Center for Genomics and Bioinformatics Core Facility are provided in the methods section.

For manuscripts utilizing custom algorithms or software that are central to the research but not yet described in published literature, software must be made available to editors and reviewers. We strongly encourage code deposition in a community repository (e.g. GitHub). See the Nature Research guidelines for submitting code & software for further information.

Data

Policy information about availability of data

All manuscripts must include a data availability statement. This statement should provide the following information, where applicable:

- Accession codes, unique identifiers, or web links for publicly available datasets
- A list of figures that have associated raw data
- A description of any restrictions on data availability

All data are included in this published article and its Supplementary Information files. The RNA-Seq data that support the findings of this study have been deposited in the NCBI Gene Expression Omnibus and are accessible through the GEO Series accession number GSE153528 (https://www.ncbi.nlm.nih.gov/geo/query/acc.cgi?

acc=GSE153528). Source data are provided with this paper.

Field-specific reporting

Please select the one below that is the best fit for your research. If you are not sure, read the appropriate sections before making your selection.

- [x] Life sciences
- [ ] Behavioural & social sciences
- [ ] Ecological, evolutionary & environmental sciences

For a reference copy of the document with all sections, see nature.com/documents/nr-reporting-summary-flat.pdf

Life sciences study design

All studies must disclose on these points even when the disclosure is negative.

Sample size	For the initial experiments described in Figure 1A-E, we performed sample-size calculation by Fisher exact test. We assumed that after LPS injection, the rate of complications is 20% in non-iron-loaded pregnancies vs 100% in high-iron pregnancies, 6 dams/group revealed significant differences in outcome with power of 0.9 and p< 0.05.<br>For all subsequent mouse experiments, sample sizes were decided based on the initial calculations and expected outcomes. Sample sizes for in vivo experiments are indicated in each figure panel.<br>For in vitro experiments, at least 3 independent experiments were performed in order to calculate statistical significance and verify results. The number of experiments performed for in vitro data are indicated in each figure panel.
Data exclusions	We did not exclude any data from analyses.
Replication	The number of replicates for each experiment are indicated in each figure panel. All attempts of replication were successful.
Randomization	Mice were randomly allocated into the experimental groups.<br>Because each pregnant mouse can have up to 12 fetuses, not all placentas/fetuses were analyzed from all pregnancies. Rather, placentas/fetal tissues were randomly selected for analyses so that multiple samples were analyzed from at least 3 or more different pregnancies.<br>For in vitro studies, randomization was not applicable to our experimental set up.
Blinding	Investigators were not blinded during the study because the investigators who generated mouse samples also analyzed those samples.

Reporting for specific materials, systems and methods

We require information from authors about some types of materials, experimental systems and methods used in many studies. Here, indicate whether each material, system or method listed is relevant to your study. If you are not sure if a list item applies to your research, read the appropriate section before selecting a response.

Materials & experimental systems

n/a	Involved in the study	
☐	☒	Antibodies
☐	☒	Eukaryotic cell lines
☒	☐	Palaeontology and archaeology
☐	☒	Animals and other organisms
☒	☐	Human research participants
☒	☐	Clinical data
☒	☐	Dual use research of concern

Methods

n/a	Involved in the study	
☒	☐	ChIP-seq
☒	☐	Flow cytometry
☒	☐	MRI-based neuroimaging

Antibodies

Antibodies used	Primary antibodies:<br>Mouse and human cleaved caspase-3 Rabbit monoclonal antibody 9664, Cell Signaling Technology, 1:1,000<br>Mouse and human total caspase-3 Rabbit polyclonal antibody 9662, Cell Signaling Technology, 1:5,000<br>Mouse and human ferritin heavy chain Rabbit monoclonal antibody 4393, Cell Signaling Technology, 1:25,000<br>Mouse and human ferritin light chain Goat polyclonal NBP1-06986, Novus Biologicals, 1:5,000<br>Mouse and human β-actin Monoclonal antibody-peroxidase A3854, Sigma, 1:50,000<br>Mouse IL-1 beta /IL-1F2 Goat polyclonal antibody AF401, R&D Systems, 1:1,000<br>Mouse cleaved caspase-1 Rabbit monoclonal antibody 89332, Cell Signaling Technology, 1:3,000<br>Human caspase-9 Mouse monoclonal antibody 9508, Cell Signaling Technology. 1:1,000<br>Mouse and human electron transport chain Total OXPHOS Rodent WB Antibody Cocktail ab110413, Abcam, 1:5,000<br>Mouse CD31/PECAM-1 Goat polyclonal antibody AF3628, Novus Biologicals, 1:1,000<br><br>Secondary antibodies:

Anti-mouse IgG HRP antibody 7076, Cell Signaling Technology, 1:5,000
Anti-rabbit IgG HRP antibody 7074, Cell Signaling Technology, 1:5,000
Anti-goat IgG HRP antibody 2354, Santa Cruz, 1:5,000
ImmPRESS horse anti-rabbit IgG HRP detection kit MP-7401, Vector Laboratories
ImmPRESS horse anti-goat IgG AP detection kit MP-5405, Vector Laboratories

Neutralizing antibodies:
Human TNFα neutralizing Rabbit monoclonal antibody 7321, Cell Signaling Technology
Mouse TNFα neutralizing Rat monoclonal antibody clone XT3.11, BioXcell
Rat IgG isotype control, anti-trinitrophenol Rat monoclonal antibody clone TNP6A7, BioXcell

Validation

All the antibodies used in the study were validated by the manufacturing companies for mouse and human specificity and for the applications described in the study. All antibodies were further validated by our laboratory via expected and predicted molecular weight by Western blotting and including controls that are known to increase or decrease expression of the proteins visualized. For example, TNF treatment is known to induce cleaved caspase-3, and iron is known to induce expression of ferritin. For all antibodies reported, optimal dilutions were determined by our laboratory for the indicated applications.

Antibody validation by the manufacturing companies are as follows:

1. Mouse and human cleaved caspase-3 Rabbit monoclonal antibody 9664, clone 5A1E, Cell Signaling Technology: Cleaved Caspase-3 Rabbit mAb detects endogenous levels of the large fragment (17/19 kDa) of activated caspase-3 resulting from cleavage adjacent to Asp175. This antibody does not recognize full length caspase-3 or other cleaved caspases. Monoclonal antibody is produced by immunizing animals with a synthetic peptide corresponding to amino-terminal residues adjacent to Asp175 of human caspase-3, and reacts with human, mouse, rat, and monkey samples which was determined by testing in at least one approved application (see https://www.cellsignal.com/datasheet.jsp?productId=9664&images=1&protocol=0)

2. Mouse and human total caspase-3 Rabbit polyclonal antibody 9662, Cell Signaling Technology: Caspase-3 Antibody detects endogenous levels of full-length caspase-3 (35 kDa). Polyclonal antibodies are produced by immunizing animals with a synthetic peptide corresponding to residues surrounding the cleavage site of human caspase-3. Antibodies are purified by protein A and peptide affinity chromatography. Antibody reacts with human, mouse, rat, and monkey samples which was determined by testing in at least one approved application (see https://www.cellsignal.com/datasheet.jsp?productId=9662&images=1&protocol=0)

3. Mouse and human ferritin heavy chain Rabbit monoclonal antibody 4393, clone D1D4, Cell Signaling Technology: FTH1 (D1D4) Rabbit mAb recognizes endogenous levels of total FTH1 protein. Monoclonal antibody is produced by immunizing animals with a synthetic peptide corresponding to residues near the carboxy terminus of human FTH1 protein. Antibody reacts with human, mouse, rat, and monkey samples which was determined by testing in at least one approved application (see https://www.cellsignal.com/datasheet.jsp?productId=4393&images=1&protocol=0).

4. Mouse and human beta-actin Monoclonal antibody-peroxidase A3854, clone Ac-15, Sigma: Mouse monoclonal anti-β-actin-peroxidase antibody specifically localizes β-actin in a wide variety of tissues and species using immunoblotting (42kDa). The antibody cross-reacts with β-Actin expressed in cells of human, bovine, sheep, pig, rabbit, cat, dog, mouse, rat, guinea pig, chicken, carp, and Hirudo medicinalis (leech) tissues, but not in Dictyostelium discoideum amoebae or Drosophila (see https://www.sigmaaldrich.com/content/dam/sigma-aldrich/docs/Sigma/Datasheet/6/a3854dat.pdf)

5. Mouse IL-1 beta /IL-1F2 Goat polyclonal antibody AF401, R&D Systems: Polyclonal goat IgG detects mouse IL-1 beta/IL-1F2 in direct ELISAs and Western blots, and neutralizes IL-1beta/IL-1F2 induced proliferation in D10.G4.1 mouse helper T cell line (see https://resources.rndsystems.com/pdfs/datasheets/af-401-na.pdf?v=20210503&_ga=2.147637303.1226532471.1620076195-1003374400.1620076195).

6. Mouse cleaved caspase-1 Rabbit monoclonal antibody 89332, clone E2G2I, Cell Signaling Technology: Cleaved Caspase-1 (Asp296) (E2G2I) Rabbit mAb recognizes endogenous levels of caspase-1 protein only when cleaved at Asp296. A non-specific band is detected at 70 kDa in some cells. Monoclonal antibody is produced by immunizing animals with a synthetic peptide corresponding to residues surrounding Asp296 of mouse caspase-1 protein. Antibody reacts with mouse samples which was determined by testing in at least one approved application (see https://www.cellsignal.com/datasheet.jsp?productId=89332&images=1&protocol=0).

7. Human caspase-9 Mouse monoclonal antibody 9508, clone C9, Cell Signaling Technology: Caspase-9 (C9) Antibody detects endogenous levels of the pro form of caspase-9 as well as cleaved fragments. Monoclonal Antibody is produced by immunizing mice with a recombinant human caspase-9 protein. Antibody reacts with human, mouse, rat, hamster, and monkey samples which was determined by testing in at least one approved application (see https://www.cellsignal.com/datasheet.jsp?productId=9508&images=1&protocol=0).

8. Mouse and human electron transport chain Total OXPHOS Rodent WB Antibody Cocktail ab110413, Abcam: Total OXPHOS Rodent WB Antibody Cocktail ab110413 is an optimized cocktail of high-quality antibodies for analyzing relative levels of OXPHOS complexes in rat or mouse mitochondria by western blot. Antibody reacts with mouse, rat, cow, human, and cynomolgus monkey (see https://www.abcam.com/total-oxphos-rodent-wb-antibody-cocktail-ab110413.pdf).

9. Mouse CD31/PECAM-1 Goat polyclonal antibody AF3628, Novus Biologicals: Detects mouse CD31/PECAM-1 in direct ELISAs and Western blots. In direct ELISAs and Western blots, approximately 10% cross-reactivity with recombinant human CD31 and recombinant porcine CD31 is observed. Detects mouse CD31 and rat CD31 in flow cytometry. Polyclonal antibody reacts with mouse and rat samples and is validated for immunostaining (see https://resources.rndsystems.com/pdfs/datasheets/af3628.pdf?_ga=2.177128037.1748988963.1620079308-1742516439.1620079307).

10. Anti-mouse IgG HRP antibody 7076, Cell Signaling Technology: Affinity purified horse anti-mouse IgG (heavy and light chain) antibody is conjugated to horseradish peroxidase(HRP) for chemiluminescent detection. This product is thoroughly validated with CST primary antibodies and will work optimally with the CST western immunoblotting protocol, ensuring accurate and reproducible results (see https://www.cellsignal.com/datasheet.jsp?productId=7076&images=1&protocol=0).

11. Anti-rabbit IgG HRP antibody 7074, Cell Signaling Technology: Designed for use with rabbit polyclonal and monoclonal antibodies, this affinity purified goat anti-rabbit IgG (heavy and light chain) antibody is conjugated to horseradish peroxidase(HRP) for chemiluminescent detection. This product is thoroughly validated with CST primary antibodies and will work optimally with the CST western immunoblotting protocol, ensuring accurate and reproducible results (see https://www.cellsignal.com/datasheet.jsp?productId=7074&images=1&protocol=0).

12. Anti-goat IgG HRP antibody 2354, Santa Cruz: mouse anti-goat IgG-HRP is an affinity purified secondary antibody raised in mouse against goat IgG and conjugated to HRP (horseradish peroxidase). mouse anti-goat IgG-HRP is recommended for detection of goat IgG by ECL Western Blotting (see https://datasheets.scbt.com/sc-2354.pdf).

13. ImmPRESS horse anti-rabbit IgG HRP detection kit MP-7401, Vector Laboratories: The ImmPRESS polymerized reporter enzyme staining system uses novel conjugation and micropolymer chemistries to create a highly sensitive, ready-to-use, one-step, non-biotin detection system for immunohistochemistry and immunocytochemistry staining. This unique micropolymer of highly active horseradish peroxidase (HRP) is attached to our affinity purified secondary antibodies (see https://vectorlabs.com/productpdf/download/file/id/1381/name/ImmPRESS%25C2%25AE_HRP_Horse_Anti-Rabbit_IgG_Polymer_Detection_Kit%252C__Peroxidase.pdf/).

14. ImmPRESS horse anti-goat IgG AP detection kit MP-5405, Vector Laboratories: The ImmPRESS polymerized reporter enzyme staining system uses novel conjugation and micropolymer chemistries to create a highly sensitive, ready-to-use, one-step detection system. This unique micropolymer of highly active enzyme (alkaline phosphatase) is attached to our affinity purified secondary antibodies (see https://vectorlabs.com/productpdf/download/file/id/1436/name/ImmPRESS%25C2%25AE-AP_Horse_Anti-Goat_IgG_Polymer_Detection_Kit%252C_Alkaline_Phosphatase.pdf/).

15. Human TNFalpha neutralizing Rabbit monoclonal antibody 7321, clone D1B4, Cell Signaling Technology: Monoclonal antibody is produced by immunizing animals with a recombinant human TNF-α protein. Monoclonal antibody is produced by immunizing animals with a recombinant human TNF-α protein (see https://www.cellsignal.com/datasheet.jsp?productId=7321&images=1&protocol=0).

16. Mouse TNFalpha neutralizing Rat monoclonal antibody clone XT3.11, BioXcell: Monoclonal antibody Purified from tissue culture supernatant in an animal free facility. The XT3.11 monoclonal antibody reacts with mouse TNFalpha in in vivo and in vitro TNFalpha neutralization and Western blot (see https://d2a7cdyquyl45u.cloudfront.net/tds-sheets/BE0058-tds.pdf).

17. Rat IgG isotype control, anti-trinitrophenol Rat monoclonal antibody clone TNP6A7, BioXcell: The TNP6A7 monoclonal antibody reacts with trinitrophenol (TNP). Because TNP is not expressed by mammals this antibody is ideal for use as an isotype-matched control for rat IgG1 antibodies in most in vivo and in vitro applications (https://d2a7cdyquyl45u.cloudfront.net/tds-sheets/BE0290-tds.pdf).

Eukaryotic cell lines

Policy information about cell lines

Cell line source(s)	Primary human umbilical vein endothelial cells (HUVECs) pooled from 10 donors were obtained from the American Type Culture Collection (ATCC, Rockefeller, MD, USA) (ATCC #PCS-100-013)
Authentication	Primary HUVECs were not authenticated in our laboratory as they were obtained directly from ATCC.
Mycoplasma contamination	Primary HUVECs were negative for mycoplasma contamination when tested by ATCC. Cells were not retested in our laboratory.
Commonly misidentified lines (See ICLAC register)	No commonly misidentified cell lines were used.

Animals and other organisms

Policy information about studies involving animals; ARRIVE guidelines recommended for reporting animal research

Laboratory animals	The following strains of animals were maintained on a 12-hour light–dark schedule in a temperature- (22–25°C) and humidity-controlled environment: wild-type C57BL/6 mice (JAX stock #000664) purchased from the Jackson Laboratory (Bar Harbor, ME, USA), hepcidin-1 knockout mice on the C57BL/6 background were originally provided by Dr. Sophie Vaulont (Paris, France) 14 years ago and backcrossed by us. Pregnant mice were all females by default and ranged from 10-24 weeks of age. Embryos of both sexes were analyzed.
Wild animals	The study did not involve wild animals.
Field-collected samples	The study did not involve field-collected samples.
Ethics oversight	All animal experiments were approved by the University of California, Los Angeles (UCLA) Institutional Animal Care and Use Committee and were carried out in accordance with the Guide for Care and Use of Laboratory Animals (National Institutes of Health, Bethesda, MD).

Note that full information on the approval of the study protocol must also be provided in the manuscript.
